# Serum Proenkephalin A Levels and Mortality After Long-Term Follow-Up in Patients with Type 2 Diabetes Mellitus (ZODIAC-32)

**DOI:** 10.1371/journal.pone.0133065

**Published:** 2015-07-28

**Authors:** Kornelis J. J. van Hateren, Gijs W. D. Landman, Jarinke F. H. Arnold, Hanneke Joosten, Klaas H. Groenier, Gerjan J. Navis, Andrea Sparwasser, Stephan J. L. Bakker, Henk J. G. Bilo, Nanne Kleefstra

**Affiliations:** 1 Diabetes Centre, Isala, Zwolle, the Netherlands; 2 Department of Internal Medicine, Gelre hospital, Apeldoorn, the Netherlands; 3 University of Groningen, University Medical Center Groningen, Department of Internal Medicine, Groningen, the Netherlands; 4 University of Groningen, University Medical Center Groningen, Department of General Practice, Groningen, the Netherlands; 5 Research Department, B.R.A.H.M.S GmbH (Thermo Fisher Scientific), Hennigsdorf/Berlin, Germany; 6 Department of Internal Medicine, Isala, Zwolle, the Netherlands; 7 Langerhans Medical Research Group, Zwolle, the Netherlands; Baylor College of Medicine, UNITED STATES

## Abstract

**Background:**

Two previous studies concluded that proenkephalin A (PENK-A) had predictive capabilities for stroke severity, recurrent myocardial infarction, heart failure and mortality in patients with stroke and myocardial infarction.

**Objectives:**

This study aimed to investigate the value of PENK-A as a biomarker for predicting mortality in patients with type 2 diabetes mellitus.

**Methods:**

Patients with type 2 diabetes mellitus were included from the prospective observational ZODIAC (Zwolle Outpatient Diabetes project Integrating Available Care) study. The present analysis incorporated two ZODIAC cohorts (1998 and 2001). Since blood was drawn for 1204 out of 1688 patients (71%), and information on relevant confounders was missing in 47 patients, the final sample comprised 1157 patients. Cox proportional hazard models were used for evaluating the relationship between PENK-A and (cardiovascular) mortality. Risk prediction capabilities were assessed with Harrell’s C statistics and the integrated discrimination improvement (IDI).

**Results:**

After a follow-up period of 14 years, 525 (45%) out of 1157 patients had died, of which 224 (43%) were attributable to cardiovascular factors. Higher Log PENK-A levels were not independently associated with increased (cardiovascular) mortality. Patients with PENK-A values in the highest tertile had a 49% (95%CI 1%-121%) higher risk of cardiovascular mortality compared to patients in the reference category (lowest tertile). C-values were not different after removing PENK-A from the Cox models and there were no significant differences in IDI values.

**Conclusions:**

The associations between PENK-A and mortality were strongly attenuated after accounting for all traditional risk factors. Furthermore, PENK-A did not seem to have additional value beyond conventional risk factors when predicting all-cause and cardiovascular mortality.

## Introduction

The opioid peptide enkephalin is found in brain and endocrine tissues and has effects on neuroendocrine and nociceptive responses [1][2]. Enkephalin expression in the hypothalamus is stimulated in response to fat consumption and is thought to inhibit nociceptive responses in the spinal cord [3][4]. Enkephalins are also expressed by pancreatic islets cells [5], and have an inhibitory effect on the insulin response through inhibiting glucose absorption [6]. Furthermore, a variety of other physiological effects have been described, for example its influence on heart rate and blood pressure [7].

As enkephalins have a very short half-life, a stable precursor fragment of enkephalin (proenkephalin A (PENK-A)) has been developed and investigated in clinical studies [8]. Given the physiological effects, enkephalins have the potential to function as biomarkers for disease prediction. Two previous studies indeed showed that PENK-A levels had prognostic capabilities in patients with stroke and myocardial infarction [9][10]. Serum levels of PENK-A were related to stroke severity, recurrent myocardial infarction, heart failure and mortality [9][10].

No previous studies with either long-term follow-up, nor studies specifically in patients with type 2 diabetes mellitus (T2DM) have been performed. We hypothesized that increased PENK-A levels are associated with cardiovascular and all-cause mortality in patients with T2DM

## Materials and Methods

### Study sample

This prospective observational study of primary care treated patients is part of the ZODIAC (Zwolle Outpatient Diabetes project Integrating Available Care) study; the design and details of which have been presented elsewhere [11]. This project started in 1998 in Zwolle, The Netherlands, and is still ongoing. Briefly, the aim of the parent study was to study the effects of two different shared care interventions in patients with T2DM. The main findings were that structured shared care with task delegation to nurses appeared feasible and could positively affect quality of care for patients with T2DM [11]. The present study is a secondary analysis of the ZODIAC study to assess the predictive capability of PENK-A, and comprises two cohorts of the ZODIAC study: one cohort started at the beginning in 1998 and the other in 2001 [12]. The first cohort contained 1143 patients and the second cohort included 973 patients, of which 427 patients were already participating in the ZODIAC study since 1998. Therefore, the combined cohort consisted of 1688 patients.

### Data collection

Baseline data consisting of a full medical history were collected in 1998 and 2001. Patients were considered to have macrovascular complications when they had a previous history of angina pectoris, myocardial infarction, percutaneous transluminal coronary angioplasty, coronary artery bypass grafting, stroke, or transient ischaemic attack. Physical and laboratory assessment data, such as blood pressure, body mass index, lipid profile, creatinine levels, HbA1c and urinary albumin-creatinine ratio, were collected annually. Blood pressure was measured twice with a Welch Allyn Sphygmomanometer in supine position after at least five minutes of rest. The mean blood pressure of two recordings was calculated for each visit.

### Measurement of proenkephalin A

Since mature enkephalins that derive from the PENK-A precursor peptide, are unstable bioactive peptides, an immunoassay has been developed that measures a stable precursor fragment of PENK-A, termed PENK-A 119–159 [8]. The abbreviation PENK-A used in this manuscript refers to this precursor fragment. PENK-A was measured using a sandwich immunoassay, in 1204 out of the 1688 (71.3%) patients using serum collected at baseline and kept frozen at -80 degrees Celsius until analysis according to the instructions of the manufacturer (B.R.A.H.M.S. GmbH, Hennigsdorf/Berlin, Germany). The functional assay sensitivity of this assay is 18.5 pmol/L (20% CV) (unpublished data).

### Clinical endpoints

There were two clinical endpoints: all-cause and cardiovascular mortality. The vital status and cause of death were retrieved from records maintained by the hospital and the general practitioners up to the end of 2012. Causes of death were coded according to The International Classification of Diseases, 9th revision (ICD-9). Cardiovascular mortality was defined as any death in which the principal cause of death was cardiovascular in nature (ICD-9 codes 390–459).

### Statistical analyses

Analyses were performed with SPSS version 18.0 (SAS Institute, Cary, NC, USA) and STATA version 12 (StataCorp, College Station, Texas USA). Continuous variables are represented as mean (± standard deviation) for normally distributed values and as median [interquartile range] for the non-normally distributed variables. Normality of the variables was examined by inspecting Q-Q plots. Univariate linear regression analyses were used to investigate whether PENK-A concentrations were associated with several clinical parameters. Due to a skewed distribution, PENK-A and serum creatinine were logarithmically transformed.

Multivariate linear regression models were used to assess the association between PENK-A and baseline characteristics. Cox proportional hazards models were used to investigate the relationship between the serum concentration PENK-A, as a continuous and a categorical (PENK-A divided into tertiles) variable, and all-cause and cardiovascular mortality with and without adjustment for selected confounders. We used 1. an unadjusted model, 2. an age- and gender-adjusted model, and 3. a model in which we additionally adjusted for the following variables: duration of diabetes, smoking (dichotomous), macrovascular complications (dichotomous), body mass index (BMI), systolic blood pressure, HbA1c, serum creatinine, cholesterol-HDL ratio and albuminuria (dichotomous). Because of missing information regarding albuminuria, BMI, smoking and duration of diabetes in 33, 2, 6 and 6 patients respectively, all analyses were performed in 1157 out of 1204 patients (96%) whose PENK-A values were determined. STATA’s ph-tests were used to test the assumption of proportional hazards for baseline predictors. Survival curves were used to illustrate the relationship between PENK-A and mortality.

For assessing the prognostic capabilities of PENK-A, we performed the following analyses stepwise. First, calibration was investigated using the Grønnesby and Borgan test assessing the goodness of fit; a nonsignificant result means an acceptable calibration [13]. Calibration is a measure of how well predicted probabilities agree with actual observed risk. Second, the Harrell’s C statistic was used to investigate the capability of each model to predict mortality and to compare how well the presence of PENK-A, in the different models used, predicted mortality [14]. The Harrell’s C value is a rank-based measure (more or less comparable with the area under the receiver operating characteristic curve). The higher the value, the better the model predicts mortality. Third, the integrated discrimination improvement (IDI) was calculated [15]. The IDI can be interpreted as the difference between model-based probabilities for events and non-events for the models with and without PENK-A. Finally, the proportion of variation explained by the survival model was estimated using R2 (D-method) [16].

In addition to investigate whether the relationship between PENK-A and mortality was affected by other clinical variables, interaction terms were added to model 3. Interaction was tested when variables were associated with PENK-A in the multivariate linear regression analyses. Interaction was evaluated as significant at a p-value of 0.10 [17].

### Ethics statement

Both cohorts of the ZODIAC study (1998 and 2001) and the informed consent procedure were approved by the local medical ethics committee of the Isala hospital, Zwolle, The Netherlands. Verbal informed consent was obtained for all patients by the participating diabetes specialist nurses and the consent was documented in the patients records. According to Dutch law, written informed consent was not necessary for this type of study in 1998 and 2001. All data were analysed anonymously.

## Results

The baseline characteristics are presented in Table 1. The median [interquartile range] PENK-A concentration was 112 [91–143] pmol/L. There were no relevant or significant differences between the final study group and those subjects without PENK-A measurements (n = 285). In the multivariate regression analyses, higher age, female gender and higher serum creatinine levels were associated with higher PENK-A levels (Table 1). Furthermore, higher HbA1c levels and BMI were related to lower PENK-A levels.

**Table 1:** Baseline characteristics and results of the multivariate linear regression models.

	Total	Tertile 1	Tertile 2	Tertile 3	Beta coefficient
Characteristic	*n = 1157*	*n = 385*	*n = 389*	*n = 383*	(95% CI)
Proenkephalin A (pmol/L)	112 [91–143]	81 [69–79]	112 [104–123]	158 [144–185]	NA
Age (years)	66.6 (± 11.6)	61.7 (± 11.4)	66.0 (± 10.5)	72.2 (± 10.4)	0.007 (0.005; 0.009)
Female sex	637 (55.1%)	189 (49.1%)	198 (50.9%)	250 (65.3%)	0.175 (0.135; 0.216)
Diabetes duration (years)	4 [2–9]	4 [2–8]	4 [2–9]	4 [2–10]	0.002 (-0.001; 0.005)
Smokers	217 (18.8%)	98 (25.5%)	63 (16.2%)	56 (14.6%)	0.000 (-0.049; 0.049)
Macrovascular complications	412 (35.6%)	119 (30.9%)	128 (32.9%)	165 (43.1%)	0.018 (-0.021; 0.058)
Body mass index (kg/m2)	29.2 (± 4.8)	30.6 (± 5.1)	29.2 (± 4.4)	27.8 (± 4.5)	-0.013 (-0.017; -0.009)
Systolic blood pressure (mmHg)	152.0 (± 23.8)	148.8 (± 24.1)	152.9 (± 23.1)	154.3 (± 23.9)	0.000 (-0.001; 0.001)
HbA1c (%) [mmol/mol]	7.2 (± 1.3) [55]	7.5 (± 1.4) [58]	7.2 (± 1.3) [55]	7.0 (± 1.3) [53]	-0.033 (-0.048; -0.018)
Serum creatinine (umol/L)	92 [82–103]	88 [79–98]	92 [83–102]	96 [86–112]	0.007 (0.006; 0.008)
Cholesterol-HDL ratio (mmol/L)	4.9 (± 1.5)	5.0 (± 1.4)	4.9 (± 1.6)	4.8 (± 1.5)	-0.002 (-0.015; 0.011)
Albuminuria present	453 (39.2%)	146 (37.9%)	156 (40.1%)	151 (39.4%)	-0.020 (-0.059; 0.019)

Data are means (± SD), medians [interquartile range] or *n* (%). One-way ANOVA, Chi square, or Kruskal-Wallis test was used where appropriate to test for differences between groups.

**Fig 1:**
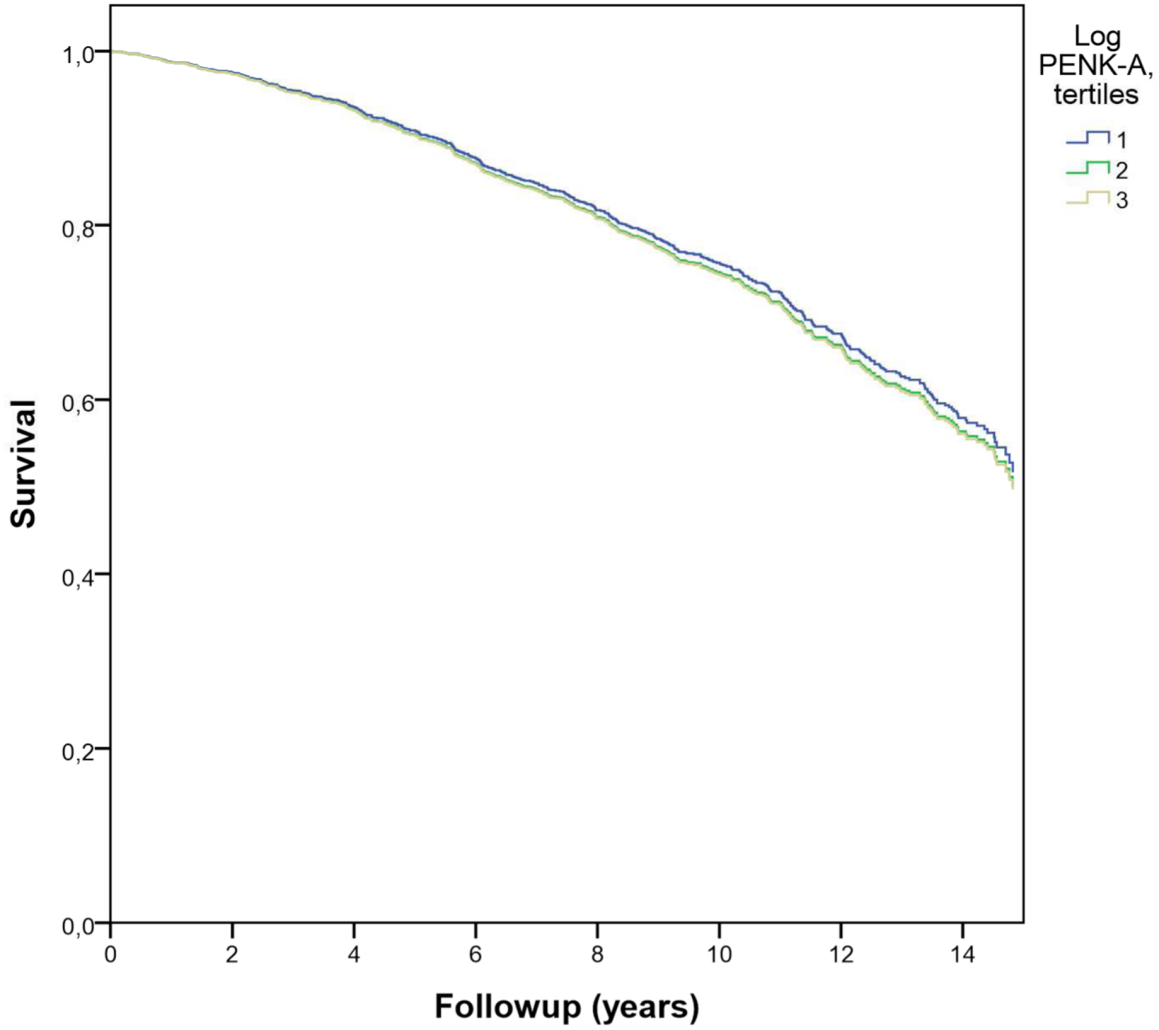
Survival curve all-cause mortality. Relationship of PENK-A, divided into tertiles, and all-cause. Tertile 1: blue line, tertile 2: green line, tertile 3: grey line.

**Fig 2:**
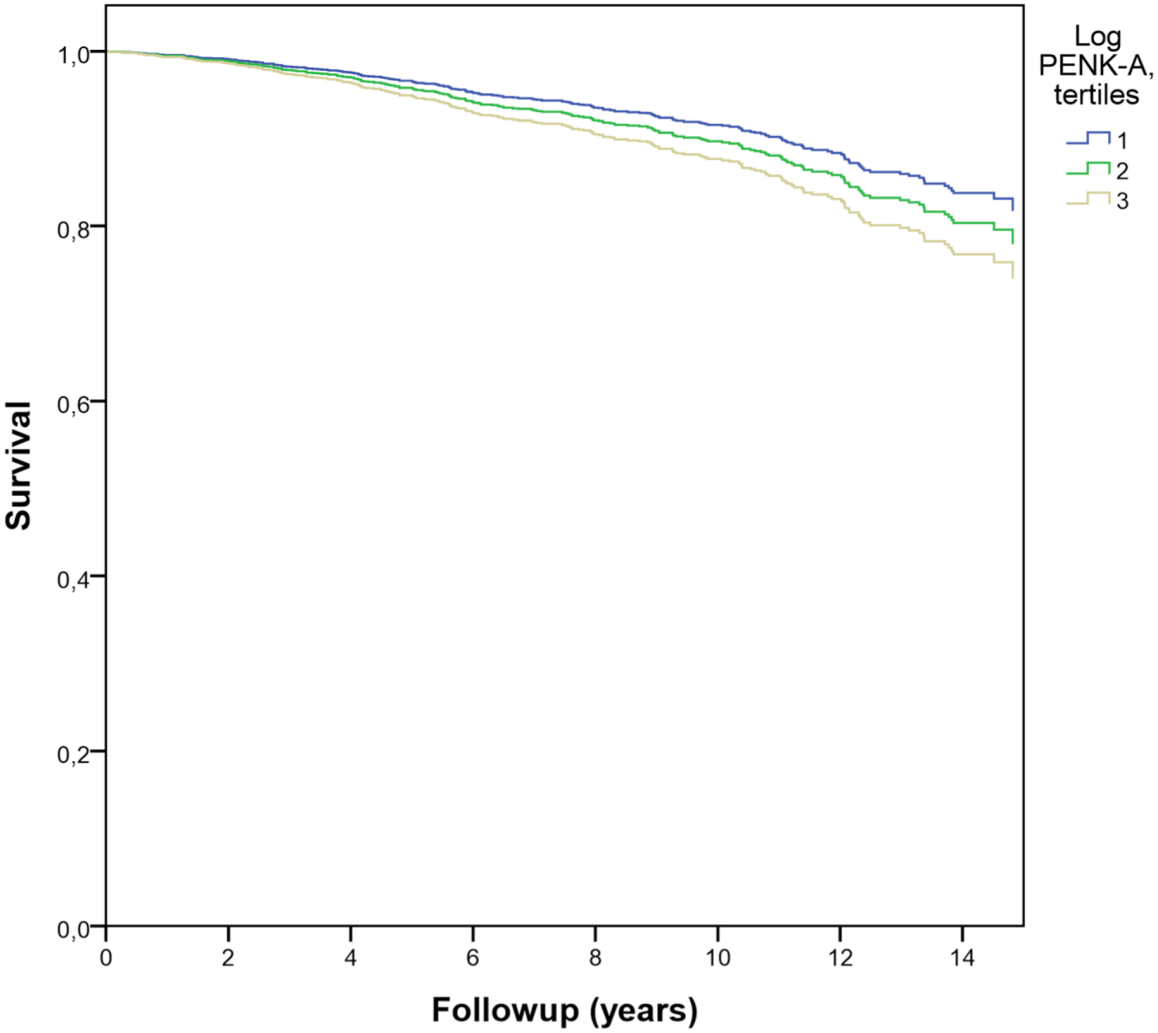
Survival curve cardiovascular mortality. Relationship of PENK-A, divided into tertiles, and cardiovascular mortality. Tertile 1: blue line, tertile 2: green line, tertile 3: grey line.

After a follow-up period of 14 years, 525 (45%) patients had died of which 224 (43%) were attributed to cardiovascular causes. A total of 13 patients were lost to follow-up (1%) and cause of death was unknown for 25 patients (5%). The median serum concentration PENK-A in the survivors was 103 [84–132] pmol/L, compared with 126 [97–159] pmol/L in the deceased patients. Results of the Cox regression analyses are presented in Table 2. Higher levels of log PENK-A were related to increased all-cause and cardiovascular mortality in the unadjusted and in the age- and gender adjusted analyses (models 1 and 2). After adjustment for all selected confounders (model 3), the relationships were not significant. Figs 1 and 2 show the survival curves of the fully adjusted models, in which PENK-A was divided into tertiles. Patients with PENK-A concentrations in the highest tertile had an increased risk of cardiovascular mortality compared to the reference category, even after adjustment for all selected confounders (Table 2 and S1 Statistical Analyses). All p-values for the ph-test were non-significant, meaning that no substantial deviations were observed.

**Table 2:** Results of the Cox regression analyses, the comparison of predictive capability for mortality as determined by the Harrell’s C statistic, and the IDI for adding the peptide to models 2 and 3, respectively.

	n	Model 1	Model 2	Model 3HR (95%CI)
		HR (95%CI)	HR (95%CI)	HR (95%CI)
**All-cause mortality**				
Tertile 1	385	1 (reference)	1 (reference)	1 (reference)
Tertile 2	389	1.33 (1.06–1.67)	0.98 (0.78–1.23)	1.05 (0.83–1.33)
Tertile 3	383	2.33 (1.88–2.89)	1.12 (0.90–1.41)	1.06 (0.83–1.35)
Log PENK-A	1157	3.27 (2.56–4.18)	1.33 (1.02–1.73)	1.09 (0.81–1.46)
Harrell’s C		0.62 (0.59–0.64)	0.77 (0.75–0.79)	0.80 (0.78–0.82)
Harrell’s C*		NA	0.77 (0.75–0.79)	0.80 (0.78–0.82)
R^2^ (95%CI)		0.10 (0.06–0.15)	0.49 (0.42–0.55)	0.58 (0.53–0.65)
IDI % (95%CI)		NA	0.1 (-0.1–0.3)	0.0 (-0.1–0.1)
***Cardiovascular mortality***				
Tertile 1	385	1 (reference)	1 (reference)	1 (reference)
Tertile 2	389	1.50 (1.03–2.21)	1.14 (0.77–1.67)	1.24 (0.84–1.82)
Tertile 3	383	3.37 (2.38–4.75)	1.77 (1.23–2.54)	1.49 (1.01–2.21)
Log PENK-A	1157	4.99 (3.44–7.24)	2.31 (1.52–3.50)	1.45 (0.91–2.30)
Harrell’s C		0.65 (0.62–0.69)	0.76 (0.73–0.79)	0.82 (0.79–0.84)
Harrell’s C*		NA	0.76 (0.73–0.79)	0.82 (0.79–0.84)
R^2^ (95%CI)		0.18 (0.10–0.28)	0.49 (0.40–0.59)	0.67 (0.60–0.76)
IDI % (95%CI)		NA	1.3 (0.6–2.0)	0.2 (-0.1–0.6)

Abbreviations: IDI, integrated discrimination improvement HR, hazard ratio; CI, confidence interval; NA, not applicable.

* Harrell’s C values for the models without PENK-A. Model 1: crude model. Model 2: adjusted for age and gender. Model 3: adjusted for age, gender, duration of diabetes, smoking (dichotomous), macrovascular complications (dichotomous), body mass index, systolic blood pressure, HbA1c, serum creatinine, cholesterol-HDL ratio and albuminuria (dichotomous).

After adding an interaction between serum creatinine and PENK-A to model 3, this interaction was only significant in the model with all-cause mortality (p = 0.019) (S2 and S3 Statistical analyses). For each quartile of serum creatinine survival plots were created illustrating the relationship between PENK-A (in quartiles) and mortality. The same plots were created for each quartile of PENK-A, in which the relationship between serum creatinine and mortality was illustrated. These plots showed that the mortality rates in patients with the highest PENK-A levels increased with higher serum creatinine values (S4 Statistical analyses). Also, the mortality rate in patients wither higher serum creatinine levels increased with higher PENK-A values. In a post-hoc explanatory analysis without serum creatinine, PENK-A was significantly and independently associated with all-cause and cardiovascular mortality (S2 and S3 Statistical analyses).

The Grønessby and Borgan test indicated that predicted probabilities corresponded well with observed risks and all models were well calibrated (data not shown). So, the number of deceased patients as estimated by the model is in line with the number of patients that actually died. According to the Harrell C statistic (Table 2), model 3 is the most capable for predicting mortality. C-values were not different after removing PENK-A from models 2 and 3. R^2 increased with adding more variables to the model. A significant IDI was only found in model 2 for cardiovascular mortality.

## Discussion

After adjustment for all traditional cardiovascular risk factors, we observed no independent association between PENK-A, as a continuous variable, and mortality. Although patients with the highest PENK-A concentrations had a 49% higher risk of cardiovascular mortality compared to patients with the lowest concentrations, the association was strongly attenuated after adjusting for all traditional risk factors. These results put conclusions from previous studies in a different perspective [9][10].

### Previous studies

Notably, the 2 previous studies that both claimed that PENK-A was promising as a biomarker, did not systematically adjust for the same important risk factors as we did in our study [9][10]. Although a relevant association between PENK-A as a continuous variable and mortality (given the width of the 95% CI, model 3) could not be excluded in this study, the associations were attenuated after adding other confounders. In our post-hoc analyses without serum creatinine, PENK-A was significantly and independently associated with all-cause and cardiovascular mortality. These results could suggest that PENK-A is a biomarker for renal function. However, Ng et al. adjusted for the estimated glomerular filtration rate (eGFR), and therefore it seems unlikely that serum creatinine is the only missing confounder. Doehner et al. only adjusted for age, stroke severity and brain lesion size, and Ng et al., although correcting for eGFR, did not include smoking, BMI, albuminuria and cholesterol in the multivariate analyses [9]. Therefore, the results of both previous could have been influenced by unmeasured confounders for which we were able to adjust for.

### Risk prediction

Although the IDI showed a significant improvement in discrimination for cardiovascular mortality prediction for the age- and gender-adjusted PENK-A levels, care must be taken to rely on these results because measures like IDI are not developed in the context of censored data. In addition, serious criticism has been published against the use of IDI and NRI indices [18][19]. Simulation results show that these measures can show predictive improvement even when non-informative predictors are added to a prediction model [20]. The addition of PENK-A to the age- and gender-adjusted models did not increase the C values. Although we cannot exclude that PENK-A has a role in risk prediction when PENK-A is studied as a single marker, there was no additional value when all traditional cardiovascular risk factors were available, at least not for mortality prediction in patients with T2DM. This study confirmed previous studies that it is difficult to achieve improvements in risk prediction by adding a biomarker to models with all conventional risk factors [21].

### Strengths and limitations

Strengths of this study were the prospective design, the high event rate (cause of death was available for 501 patients out of 525 deceased patients) and the long follow-up period. This study also had several important limitations. Firstly, the observational design prevented us from drawing conclusions on causality. Secondly, the serum concentration of PENK-A was assessed in 71% of the total study population, which could have led to selection bias. However, no relevant differences were observed in baseline variables between the final study group and those subjects without PENK-A measurements (n = 285). Furthermore, missing of PENK-A data was related to mortality and this could have led to an underestimation of the effects of PENK-A on mortality (HR all-cause mortality 1.20 (95% confidence interval 1.00–1.44)). Furthermore, PENK-A was measured only once, therefore potential daily variability in concentration or measurement errors could not be accounted for.

## Conclusions

Serum PENK-A levels were not independently associated with all-cause mortality in patients with type 2 diabetes mellitus, and its association with cardiovascular mortality was strongly attenuated after accounting for all traditional risk factors. Furthermore, PENK-A did not seem to have additional value beyond conventional risk factors when predicting all-cause and cardiovascular mortality. The results of this study show that is plausible that the results from two previous studies could have been influenced by residual confounding. Future studies assessing the prognostic capabilities of PENK-A should systematically account for all important cardiovascular risk factors.

## Supporting Information

S1 Statistical Analyses Results PENK-A in tertiles and survival curves. (PDF)

S2 Statistical Analyses Results All-cause mortality. (PDF)

S3 Statistical Analyses Results Cardiovascular mortality. (PDF)

S4 Statistical Analyses Plots illustrating interaction PENK-A and creatinine. (PDF)
