# Supplementary material for: Serum Proenkephalin A Levels and Mortality After Long-Term Follow-Up in Patients with Type 2 Diabetes Mellitus (ZODIAC-32)
Source: PLoS One. 2015 Jul 28;10(7):e0133065. doi: 10.1371/journal.pone.0133065 (PMC4517864; doi:10.1371/journal.pone.0133065)
Supplement: S1 Statistical Analyses — (PDF) [file pone.0133065.s001.pdf]

```
COXREG Followup
  /STATUS=Both_Levend(1)
  /PATTERN BY PENKA_tertielen
  /CONTRAST (PENKA_tertielen)=Indicator
  /METHOD=ENTER Both_Leeftijd Both_geslacht Both_BMI Both_roken_baseline Bo
th_RRs Both_HbA1c Both_chol_HDL_ratio Both_creat Both_DMduur Both_albuminur
ie Both_MVC PENKA_tertielen
  /PLOT SURVIVAL
  /PRINT=CI(95)
  /CRITERIA=PIN(.05) POUT(.10) ITERATE(20).
```

Cox Regression

Notes

Output Created		17-sep-2014 12:24:29
Comments		
Input	Data	C:\Users\Hans van Hateren\Documents\Documents\Onderzoek - SPSS bestanden\ZODIAC\ZODIAC-32 PENK\Werkbestand_PENKA_20130405_alleen1157pr_updatemortaliteit.sav
	Active Dataset	DataSet1
	Filter	<none>
	Weight	<none>
	Split File	<none>
	N of Rows in Working Data File	1157
Missing Value Handling	Definition of Missing	User-defined missing values are treated as missing.
Syntax		COXREG Followup /STATUS=Both_Levend(1) /PATTERN BY PENKA_tertielen /CONTRAST (PENKA_tertielen) =Indicator /METHOD=ENTER Both_Leeftijd Both_geslacht Both_BMI Both_roken_baseline Both_RRs Both_HbA1c Both_chol_HDL_ratio Both_creat Both_DMduur Both_albuminurie Both_MVC PENKA_tertielen /PLOT SURVIVAL /PRINT=CI(95) /CRITERIA=PIN(.05) POUT(.10) ITERATE(20).
Resources	Processor Time	00:00:00,406
	Elapsed Time	00:00:00,416

```
[DataSet1] C:\Users\Hans van Hateren\Documents\Documents\Onderzoek - SPSS b
estanden\ZODIAC\ZODIAC-32 PENK\Werkbestand_PENKA_20130405_alleen1157pr_upda
temortaliteit.sav
```

Case Processing Summary

		N	Percent
Cases available in analysis	Event[a]	525	45,4%
	Censored	632	54,6%
	Total	1157	100,0%
Cases dropped	Cases with missing values	0	,0%
	Cases with negative time	0	,0%
	Censored cases before the earliest event in a stratum	0	,0%
	Total	0	,0%
Total		1157	100,0%

a. Dependent Variable: Followup

Categorical Variable Codings[b]

		Frequency	(1)	(2)
PENKA_tertielen[a]	1	385	1	0
	2	389	0	1
	3	383	0	0

a. Indicator Parameter Coding

b. Category variable: PENKA_tertielen (penka log verdeeld in tertielen)

Block 0: Beginning Block

Omnibus Tests of Model Coefficients

-2 Log Likelihood
7004,412

Block 1: Method = Enter

Omnibus Tests of Model Coefficients[a]

-2 Log Likelihood	Overall (score)			Change From Previous Step		
	Chi-square	df	Sig.	Chi-square	df	Sig.
6367,634	611,068	13	,000	636,778	13	,000

a. Beginning Block Number 1. Method = Enter

Omnibus Tests of Model Coefficients[a]

Change From Previous Block		
Chi-square	df	Sig.
636,778	13	,000

a. Beginning Block Number 1. Method = Enter

Variables in the Equation

	B	SE	Wald	df	Sig.	Exp(B)
Both_Leeftijd	,103	,006	313,715	1	,000	1,108
Both_geslacht	-,103	,102	1,017	1	,313	,902
Both_BMI	,002	,011	,032	1	,858	1,002
Both_roken_baseline	,682	,119	33,131	1	,000	1,979
Both_RRs	-,004	,002	4,381	1	,036	,996
Both_HbA1c	,094	,037	6,504	1	,011	1,099
Both_chol_HDL_ratio	-,008	,032	,058	1	,810	,992
Both_creat	,009	,002	17,606	1	,000	1,009
Both_DMduur	,016	,006	6,890	1	,009	1,016
Both_albuminurie	,550	,094	34,083	1	,000	1,734
Both_MVC	,408	,092	19,846	1	,000	1,504
PENKA_tertielen			,246	2	,884	
PENKA_tertielen(1)	-,058	,124	,218	1	,641	,944
PENKA_tertielen(2)	-,010	,112	,007	1	,932	,990

Variables in the Equation

	95,0% CI for Exp(B)	
	Lower	Upper
Both_Leeftijd	1,096	1,121
Both_geslacht	,738	1,102
Both_BMI	,981	1,023
Both_roken_baseline	1,568	2,496
Both_RRs	,991	1,000
Both_HbA1c	1,022	1,182
Both_chol_HDL_ratio	,932	1,056
Both_creat	1,005	1,014
Both_DMduur	1,004	1,027
Both_albuminurie	1,441	2,086
Both_MVC	1,257	1,800
PENKA_tertielen		
PENKA_tertielen(1)	,740	1,204
PENKA_tertielen(2)	,795	1,234

Covariate Means and Pattern Values

	Mean	Pattern		
		1	2	3
Both_Leeftijd	66,602	66,602	66,602	66,602
Both_geslacht	1,551	1,551	1,551	1,551
Both_BMI	29,205	29,205	29,205	29,205
Both_roken_baseline	1,188	1,188	1,188	1,188
Both_RRs	152,038	152,038	152,038	152,038
Both_HbA1c	7,245	7,245	7,245	7,245
Both_chol_HDL_ratio	4,898	4,898	4,898	4,898
Both_creat	94,838	94,838	94,838	94,838
Both_DMduur	6,582	6,582	6,582	6,582
Both_albuminurie	1,392	1,392	1,392	1,392
Both_MVC	,356	,356	,356	,356
PENKA_tertielen(1)	,333	1,000	,000	,000
PENKA_tertielen(2)	,336	,000	1,000	,000

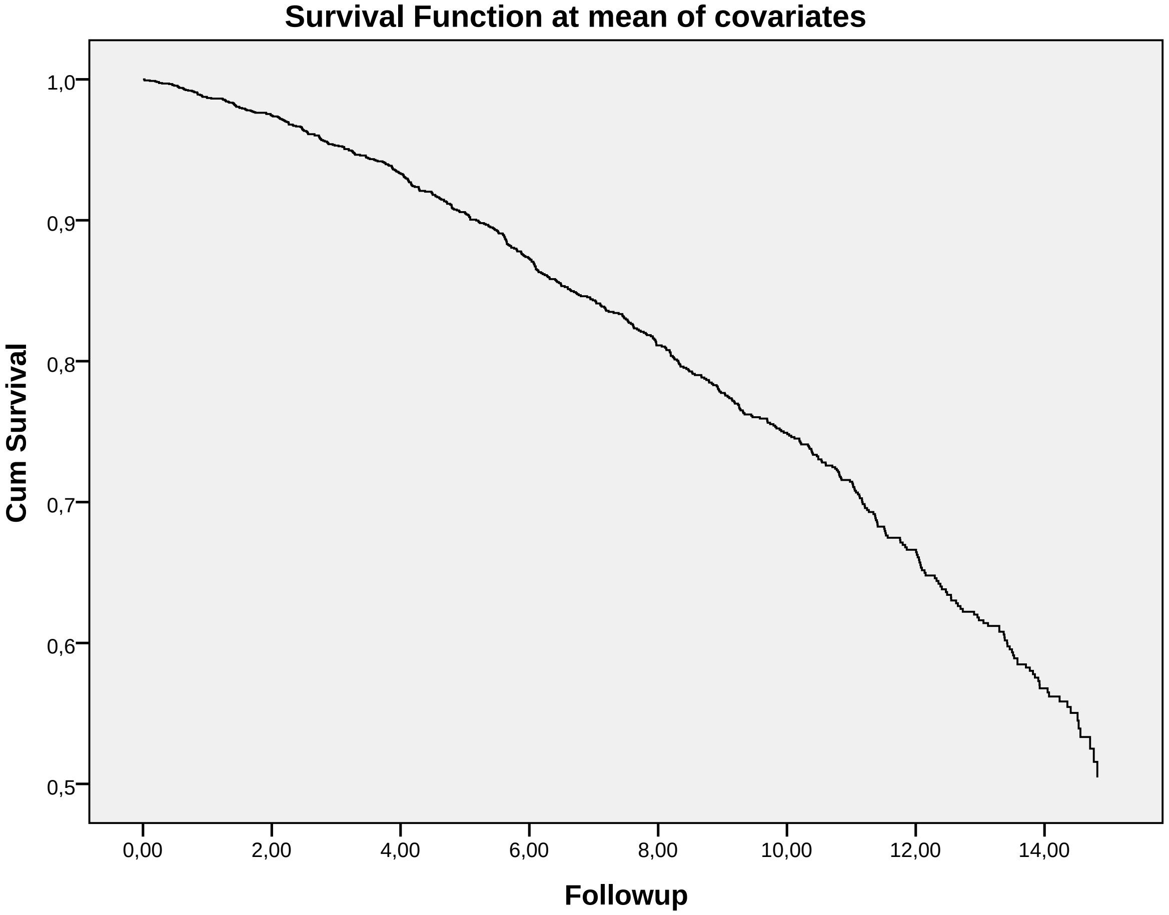

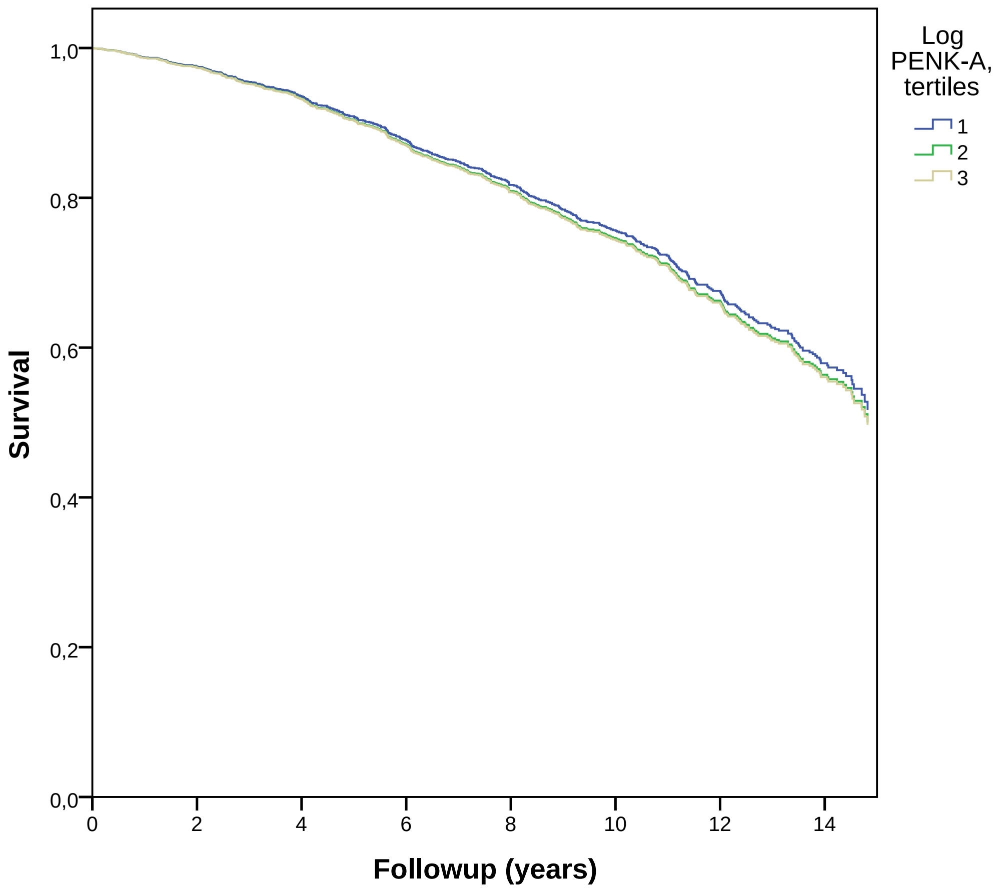


```
COXREG Followup
  /STATUS=Both_CVdeath_missing_0(1)
  /PATTERN BY PENKA_tertielen
  /CONTRAST (PENKA_tertielen)=Indicator
  /METHOD=ENTER Both_Leeftijd Both_geslacht Both_BMI Both_roken_baseline Bo
th_RRs Both_HbA1c Both_chol_HDL_ratio Both_creat Both_DMduur Both_albuminur
ie Both_MVC PENKA_tertielen
  /PLOT SURVIVAL
  /PRINT=CI(95)
  /CRITERIA=PIN(.05) POUT(.10) ITERATE(20).
```

Cox Regression

Notes

Output Created		17-sep-2014 12:24:41
Comments		
Input	Data	C:\Users\Hans van Hateren\Documents\Documents\Onderzoek - SPSS bestanden\ZODIAC\ZODIAC-32 PENK\Werkbestand_PENKA_20130405_alleen1157pr_updatemortaliteit.sav
	Active Dataset	DataSet1
	Filter	<none>
	Weight	<none>
	Split File	<none>
	N of Rows in Working Data File	1157
Missing Value Handling	Definition of Missing	User-defined missing values are treated as missing.
Syntax		COXREG Followup<br>/STATUS=Both_CVdeath_missing_0(1)<br>/PATTERN BY PENKA_tertielen<br>/CONTRAST (PENKA_tertielen)=Indicator<br>/METHOD=ENTER Both_Leeftijd Both_geslacht Both_BMI Both_roken_baseline Both_RRs Both_HbA1c Both_chol_HDL_ratio Both_creat Both_DMduur Both_albuminurie Both_MVC PENKA_tertielen<br>/PLOT SURVIVAL<br>/PRINT=CI(95)<br>/CRITERIA=PIN(.05) POUT(.10) ITERATE(20).
Resources	Processor Time	00:00:00,343
	Elapsed Time	00:00:00,337

```
[DataSet1] C:\Users\Hans van Hateren\Documents\Documents\Onderzoek - SPSS bestanden\ZODIAC\ZODIAC-32 PENK\Werkbestand_PENKA_20130405_alleen1157pr_updatemortaliteit.sav
```

Case Processing Summary

		N	Percent
Cases available in analysis	Event[a]	224	19,4%
	Censored	933	80,6%
	Total	1157	100,0%
Cases dropped	Cases with missing values	0	,0%
	Cases with negative time	0	,0%
	Censored cases before the earliest event in a stratum	0	,0%
	Total	0	,0%
Total		1157	100,0%

a. Dependent Variable: Followup

Categorical Variable Codings[b]

		Frequency	(1)	(2)
PENKA_tertielen[a]	1	385	1	0
	2	389	0	1
	3	383	0	0

a. Indicator Parameter Coding

b. Category variable: PENKA_tertielen (penka log verdeeld in tertielen)

Block 0: Beginning Block

Omnibus Tests of Model Coefficients

-2 Log Likelihood
3004,647

Block 1: Method = Enter

Omnibus Tests of Model Coefficients[a]

-2 Log Likelihood	Overall (score)			Change From Previous Step		
	Chi-square	df	Sig.	Chi-square	df	Sig.
2663,253	365,681	13	,000	341,394	13	,000

a. Beginning Block Number 1. Method = Enter

Omnibus Tests of Model Coefficients[a]

Change From Previous Block		
Chi-square	df	Sig.
341,394	13	,000

a. Beginning Block Number 1. Method = Enter

Variables in the Equation

	B	SE	Wald	df	Sig.	Exp(B)
Both_Leeftijd	,089	,009	100,835	1	,000	1,094
Both_geslacht	,004	,154	,001	1	,978	1,004
Both_BMI	-,001	,016	,006	1	,938	,999
Both_roken_baseline	,907	,176	26,568	1	,000	2,477
Both_RRs	-,002	,003	,311	1	,577	,998
Both_HbA1c	,154	,055	7,735	1	,005	1,166
Both_chol_HDL_ratio	-,019	,048	,156	1	,693	,981
Both_creat	,015	,003	27,357	1	,000	1,015
Both_DMduur	,020	,008	5,665	1	,017	1,020
Both_albuminurie	,693	,148	22,017	1	,000	2,000
Both_MVC	,667	,142	22,219	1	,000	1,949
PENKA_tertielen			4,119	2	,128	
PENKA_tertielen(1)	-,401	,199	4,064	1	,044	,670
PENKA_tertielen(2)	-,190	,173	1,211	1	,271	,827

Variables in the Equation

	95,0% CI for Exp(B)	
	Lower	Upper
Both_Leeftijd	1,075	1,113
Both_geslacht	,743	1,358
Both_BMI	,967	1,031
Both_roken_baseline	1,754	3,497
Both_RRs	,992	1,005
Both_HbA1c	1,047	1,300
Both_chol_HDL_ratio	,893	1,078
Both_creat	1,009	1,020
Both_DMduur	1,004	1,037
Both_albuminurie	1,497	2,671
Both_MVC	1,477	2,572
PENKA_tertielen		
PENKA_tertielen(1)	,454	,989
PENKA_tertielen(2)	,590	1,160

Covariate Means and Pattern Values

	Mean	Pattern 1	Pattern 2	Pattern 3
Both_Leeftijd	66,602	66,602	66,602	66,602
Both_geslacht	1,551	1,551	1,551	1,551
Both_BMI	29,205	29,205	29,205	29,205
Both_roken_baseline	1,188	1,188	1,188	1,188
Both_RRs	152,038	152,038	152,038	152,038
Both_HbA1c	7,245	7,245	7,245	7,245
Both_chol_HDL_ratio	4,898	4,898	4,898	4,898
Both_creat	94,838	94,838	94,838	94,838
Both_DMduur	6,582	6,582	6,582	6,582
Both_albuminurie	1,392	1,392	1,392	1,392
Both_MVC	,356	,356	,356	,356
PENKA_tertielen(1)	,333	1,000	,000	,000
PENKA_tertielen(2)	,336	,000	1,000	,000

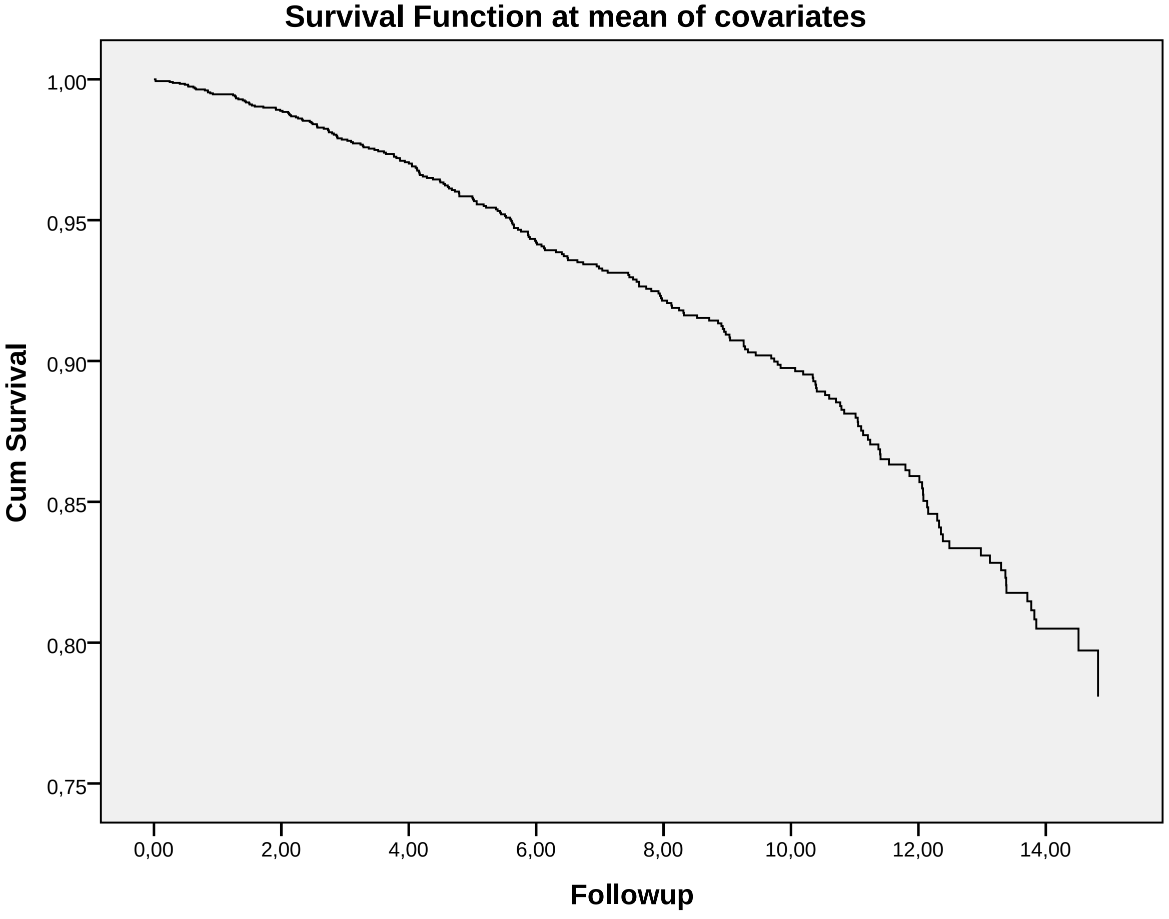

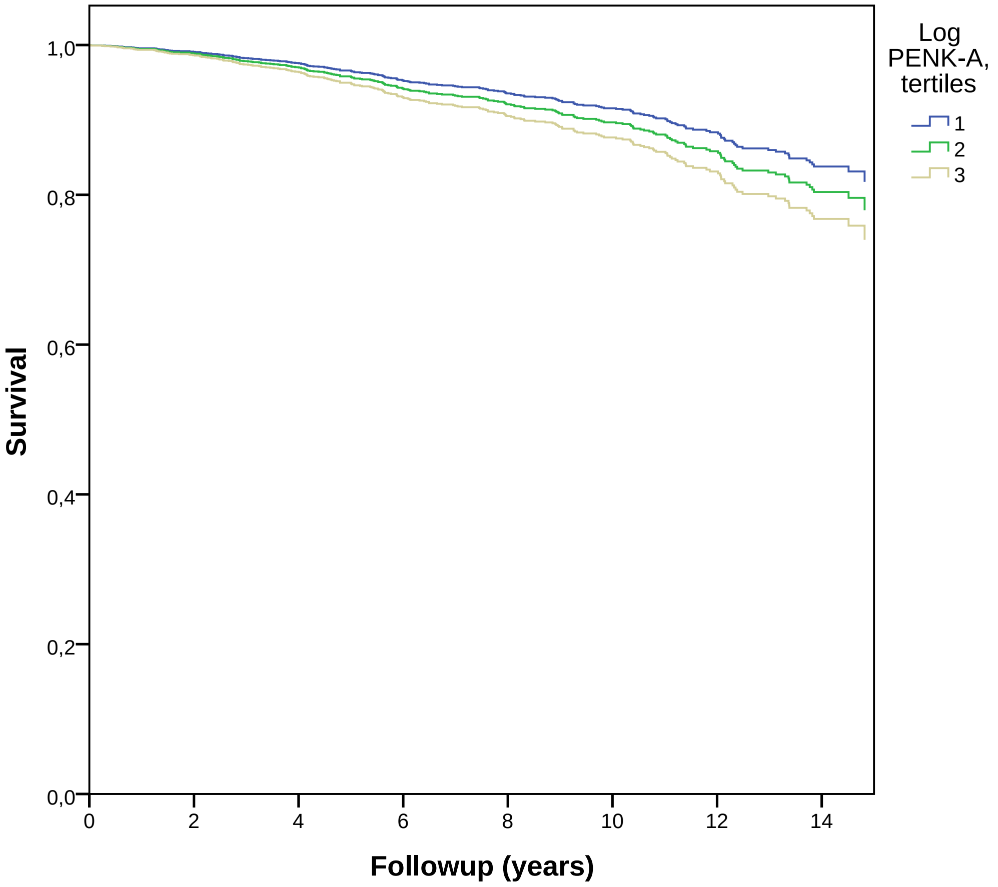
Survival
1,0
0,8
0,6
0,4
0,2
0,0
0
2
4
6
8
10
12
14
Followup (years)
Log PENK-A, tertiles
1
2
3
