# Supplementary material for: Serum Proenkephalin A Levels and Mortality After Long-Term Follow-Up in Patients with Type 2 Diabetes Mellitus (ZODIAC-32)
Source: PLoS One. 2015 Jul 28;10(7):e0133065. doi: 10.1371/journal.pone.0133065 (PMC4517864; doi:10.1371/journal.pone.0133065)
Supplement: S2 Statistical Analyses — (PDF) [file pone.0133065.s002.pdf]

```
-------------------------------------------------------------------------------------
      name:  All causes mortality
       log:  C:\Users\Groenier\Documents\Data\Diabetes\Statistiek\Arnold PENKA\Log_Pen
> ka_20130405_all_causes_update_FU.log
  log type:  text
 opened on:   3 Jun 2014, 16:39:09


. stset followup, failure(both_levend==1)

     failure event:  both_levend == 1
obs. time interval:  (0, followup]
 exit on or before:  failure

------------------------------------------------------------------------------
     1157  total obs.
        0  exclusions
------------------------------------------------------------------------------
     1157  obs. remaining, representing
      525  failures in single record/single failure data
 11602.86  total analysis time at risk, at risk from t =         0
                             earliest observed entry t =         0
                                  last observed exit t =  16.06571

.
. stcox log_both_penkapmoll, mgale(ma) schoenfeld(sch_*) scaledsch(sca_*) basesurv(bas
> e)

         failure _d:  both_levend == 1
   analysis time _t:  followup

Iteration 0:   log likelihood = -3502.2061
Iteration 1:   log likelihood = -3457.1001
Iteration 2:   log likelihood =  -3457.015
Refining estimates:
Iteration 0:   log likelihood =  -3457.015

Cox regression -- Breslow method for ties

No. of subjects =         1157                     Number of obs   =      1157
No. of failures =          525
Time at risk    =  11602.86106
                                                   LR chi2(1)      =     90.38
Log likelihood  =    -3457.015                     Prob > chi2     =    0.0000

------------------------------------------------------------------------------------
                 _t | Haz. Ratio   Std. Err.      z    P>|z|     [95% Conf. Interval]
--------------------+---------------------------------------------------------------
log_both_penkapmoll |    3.26958   .4089905     9.47   0.000     2.558677       4.178
------------------------------------------------------------------------------------

. estat ic

-----------------------------------------------------------------------------
       Model |    Obs    ll(null)   ll(model)     df          AIC         BIC
-------------+---------------------------------------------------------------
           . |   1157   -3502.206   -3457.015      1      6916.03    6921.084
-----------------------------------------------------------------------------
               Note:  N=Obs used in calculating BIC; see [R] BIC note

. estat concordance, gh se

         failure _d:  both_levend == 1
   analysis time _t:  followup

  Gonen and Heller's K concordance statistic

  Number of subjects (N)       =     1157

          Gonen and Heller's K =    .6155
                     Somers' D =    .2309
            Gonen's smoothed K =    .6154
                 Asymptotic SE =   .01113


. stcoxgof, group(10)
```

```
Goodness-of-fit test for the inclusion of design variables based on 10 quantiles of ri
> sk
(Added variables version of the Groennesby and Borgan test)

Score test                                             chi2(9)     =     6.785
                                                       Prob > chi2 =    0.6595


Likelihood-ratio test                                  LR chi2(9)  =     6.497
                                                       Prob > chi2 =    0.6893

(Table  collapsed on quantiles of linear predictor)

--------------------------------------------------------------------------------
Quantile  |
of Risk   |   Observed      Expected          z          p-Norm    Observations
----------+---------------------------------------------------------------------
        1 |        37        27.336        1.848          .065           116
        2 |        37        39.939        -.465          .642           117
        3 |        41        42.074        -.166          .869           117
        4 |        46        52.023        -.835          .404           129
        5 |        50        51.513        -.211          .833           113
        6 |        49        44.829         .623          .533           104
        7 |        54        62.551       -1.081           .28           120
        8 |        57        58.917         -.25          .803           110
        9 |        70        69.254          .09          .929           116
       10 |        84        76.565          .85          .395           115
          |
    Total |       525           525                                     1157
--------------------------------------------------------------------------------


. somersd _t invhr if _st==1, cenind(censind) tdist transf(c)
Somers' D with variable: _t
Transformation: Harrell's c
Valid observations: 1157
Degrees of freedom: 1156

Symmetric 95% CI for Harrell's c
------------------------------------------------------------------------------
             |              Jackknife
          _t |      Coef.   Std. Err.      t    P>|t|     [95% Conf. Interval]
-------------+----------------------------------------------------------------
       invhr |   .6172743    .012861    48.00   0.000     .5920409    .6425078
------------------------------------------------------------------------------

. drop hr invhr censind

. str2ph stcox log_both_penkapmoll, bootreps(1000) adjust
..........

R^2 (explained variation): Cox model

   Obs    Events   Adj. R^2    Boot. SE   95% conf. interval
------------------------------------------------------------
  1157      525    0.101393    0.020812   0.062757  0.145008
------------------------------------------------------------

.
```

```
. stset followup, failure(both_levend==1)

     failure event:  both_levend == 1
obs. time interval:  (0, followup]
 exit on or before:  failure

------------------------------------------------------------------------------
     1157  total obs.
        0  exclusions
------------------------------------------------------------------------------
     1157  obs. remaining, representing
      525  failures in single record/single failure data
 11602.86  total analysis time at risk, at risk from t =         0
                             earliest observed entry t =         0
                                  last observed exit t =  16.06571

.
. stcox log_both_penkapmoll both_geslacht both_leeftijd, schoenfeld(sch_*) mgale(ma) s
> caledsch(sca_*) basesurv(base)

         failure _d:  both_levend == 1
   analysis time _t:  followup

Iteration 0:   log likelihood = -3502.2061
Iteration 1:   log likelihood = -3263.6758
Iteration 2:   log likelihood = -3254.1665
Iteration 3:   log likelihood = -3254.1438
Refining estimates:
Iteration 0:   log likelihood = -3254.1438

Cox regression -- Breslow method for ties

No. of subjects =         1157                     Number of obs   =      1157
No. of failures =          525
Time at risk    =  11602.86106
                                                   LR chi2(3)      =    496.12
Log likelihood  =   -3254.1438                     Prob > chi2     =    0.0000

-------------------------------------------------------------------------------------
                 _t | Haz. Ratio   Std. Err.      z    P>|z|     [95% Conf. Interval]
--------------------+----------------------------------------------------------------
log_both_penkapmoll |   1.326681   .1793185     2.09   0.036     1.017924     1.72909
      both_geslacht |   .6076759   .0552156    -5.48   0.000     .5085443    .7261314
      both_leeftijd |   1.108299   .0061069    18.66   0.000     1.096395    1.120334
-------------------------------------------------------------------------------------

. estat ic

-----------------------------------------------------------------------------
       Model |    Obs    ll(null)   ll(model)     df          AIC         BIC
-------------+---------------------------------------------------------------
           . |   1157   -3502.206   -3254.144      3     6514.288    6529.448
-----------------------------------------------------------------------------
               Note:  N=Obs used in calculating BIC; see [R] BIC note

. estat concordance, gh se

         failure _d:  both_levend == 1
   analysis time _t:  followup

  Gonen and Heller's K concordance statistic

  Number of subjects (N)       =     1157

          Gonen and Heller's K =    .7558
                     Somers' D =    .5115
            Gonen's smoothed K =    .7556
                 Asymptotic SE =  .008102


. stcoxgof, group(10)

Goodness-of-fit test for the inclusion of design variables based on 10 quantiles of ri
> sk
(Added variables version of the Groennesby and Borgan test)
```

```
Score test                                                     chi2(9)     =    18.246
                                                               Prob > chi2 =    0.0324


Likelihood-ratio test                                          LR chi2(9)  =    17.219
                                                               Prob > chi2 =    0.0454

(Table  collapsed on quantiles of linear predictor)

--------------------------------------------------------------------------------
Quantile  |
of Risk   |  Observed      Expected          z           p-Norm    Observations
----------+---------------------------------------------------------------------
        1 |        13         6.299         2.67           .008           116
        2 |        16        14.886         .289           .773           116
        3 |        25         22.99         .419           .675           116
        4 |        29        32.205        -.565           .572           115
        5 |        31        47.137        -2.35           .019           116
        6 |        50        59.712       -1.257           .209           116
        7 |        76        72.673          .39           .696           115
        8 |        85        70.286        1.755           .079           116
        9 |        92        89.666         .247           .805           116
       10 |       108       109.146         -.11           .913           115
          |
    Total |       525           525                                      1157
--------------------------------------------------------------------------------


. somersd _t invhr if _st==1, cenind(censind) tdist transf(c)
Somers' D with variable: _t
Transformation: Harrell's c
Valid observations: 1157
Degrees of freedom: 1156

Symmetric 95% CI for Harrell's c
------------------------------------------------------------------------------
             |            Jackknife
          _t |      Coef.   Std. Err.      t    P>|t|     [95% Conf. Interval]
-------------+----------------------------------------------------------------
       invhr |   .7658052   .0102715    74.56   0.000     .7456523    .7859582
------------------------------------------------------------------------------

. drop hr invhr censind

. str2ph stcox log_both_penkapmoll both_geslacht both_leeftijd, bootreps(1000) adjust
..........

R^2 (explained variation): Cox model

   Obs    Events   Adj. R^2    Boot. SE   95% conf. interval
------------------------------------------------------------
  1157      525    0.486453    0.031727   0.424984  0.552413
------------------------------------------------------------
```

```
. stset followup, failure(both_levend==1)

     failure event:  both_levend == 1
obs. time interval:  (0, followup]
 exit on or before:  failure

------------------------------------------------------------------------------
     1157  total obs.
        0  exclusions
------------------------------------------------------------------------------
     1157  obs. remaining, representing
      525  failures in single record/single failure data
 11602.86  total analysis time at risk, at risk from t =         0
                             earliest observed entry t =         0
                                  last observed exit t =  16.06571

.
. stcox both_geslacht both_leeftijd, schoenfeld(sch_*) mgale(ma) scaledsch(sca_*) base
> surv(base)

         failure _d:  both_levend == 1
   analysis time _t:  followup

Iteration 0:   log likelihood = -3502.2061
Iteration 1:   log likelihood = -3266.2947
Iteration 2:   log likelihood = -3256.3828
Iteration 3:   log likelihood = -3256.3588
Refining estimates:
Iteration 0:   log likelihood = -3256.3588

Cox regression -- Breslow method for ties

No. of subjects =         1157                     Number of obs   =      1157
No. of failures =          525
Time at risk    =  11602.86106
                                                   LR chi2(2)      =    491.69
Log likelihood  =   -3256.3588                     Prob > chi2     =    0.0000

-------------------------------------------------------------------------------
           _t | Haz. Ratio   Std. Err.      z    P>|z|     [95% Conf. Interval]
--------------+----------------------------------------------------------------
both_geslacht |   .6248316   .0560924    -5.24   0.000     .5240213    .7450356
both_leeftijd |   1.112421   .0058098    20.40   0.000     1.101092    1.123867
-------------------------------------------------------------------------------

. estat ic

-----------------------------------------------------------------------------
       Model |    Obs    ll(null)   ll(model)     df          AIC         BIC
-------------+---------------------------------------------------------------
           . |   1157   -3502.206   -3256.359      2     6516.718    6526.825
-----------------------------------------------------------------------------
               Note:  N=Obs used in calculating BIC; see [R] BIC note

. estat concordance, gh se

         failure _d:  both_levend == 1
   analysis time _t:  followup

  Gonen and Heller's K concordance statistic

  Number of subjects (N)       =     1157

          Gonen and Heller's K =    .7553
                     Somers' D =    .5105
            Gonen's smoothed K =    .7551
                 Asymptotic SE =  .008149

. stcoxgof, group(10)

Goodness-of-fit test for the inclusion of design variables based on 10 quantiles of ri
> sk
(Added variables version of the Groennesby and Borgan test)

Score test                                             chi2(9)     =    15.354
                                                       Prob > chi2 =    0.0817
```

```
Likelihood-ratio test                                  LR chi2(9)  =    13.610
                                                       Prob > chi2 =    0.1369

(Table  collapsed on quantiles of linear predictor)

--------------------------------------------------------------------------------
Quantile  |
of Risk   |   Observed      Expected          z           p-Norm    Observations
----------+---------------------------------------------------------------------
        1 |        13         6.089          2.8            .005             116
        2 |        19        15.641          .849           .396             121
        3 |        23        26.464         -.673           .501             125
        4 |        25        32.844        -1.369           .171             109
        5 |        37        44.683        -1.149            .25             112
        6 |        57        65.231        -1.019           .308             124
        7 |        71        67.364          .443           .658             110
        8 |        77        67.463         1.161           .246             109
        9 |        99         92.99          .623           .533             121
       10 |       104        106.23         -.216           .829             110
          |
    Total |       525           525                                         1157
--------------------------------------------------------------------------------


. somersd _t invhr if _st==1, cenind(censind) tdist transf(c)
Somers' D with variable: _t
Transformation: Harrell's c
Valid observations: 1157
Degrees of freedom: 1156

Symmetric 95% CI for Harrell's c
------------------------------------------------------------------------------
             |              Jackknife
          _t |      Coef.   Std. Err.      t    P>|t|     [95% Conf. Interval]
-------------+----------------------------------------------------------------
       invhr |   .7659504   .0102377    74.82   0.000     .7458638     .786037
------------------------------------------------------------------------------

. drop hr invhr censind

. str2ph stcox both_geslacht both_leeftijd, bootreps(1000) adjust
..........

R^2 (explained variation): Cox model

   Obs    Events   Adj. R^2    Boot. SE   95% conf. interval
------------------------------------------------------------
  1157      525    0.483768    0.032274   0.418391  0.545469
------------------------------------------------------------

.
. idi both_levend both_geslacht both_leeftijd, prvars(log_both_penkapmoll)

----------------------------------------------------
      IDI |     Estimate     Std. Err.       P-value
----------+-----------------------------------------
          |      0.00062       0.00096       0.52295
----------------------------------------------------
```

```
. nri3 both_levend both_geslacht both_leeftijd, prvars(log_both_penkapmoll) cut(10 20
> 30)

------------------------------------------------------------------
      NRI |     Estimate     Std. Err.             Z       P-value
----------+-------------------------------------------------------
          |      0.00850       0.01113       0.76326       0.44531
------------------------------------------------------------------

------------------------------------------------------------
both_leve |
nd and    |
Establish |
ed risk   |    Established risk factors + new predictors
factors   |     <10%    10-20%  20 - 30%     >=30%     Total
----------+-------------------------------------------------
1         |
     <10% |       17         1                            18
   10-20% |                 18                            18
 20 - 30% |                  4        27                  31
    >=30% |                            5       453       458
          |
    Total |       17        23        32       453       525
----------+-------------------------------------------------
0         |
     <10% |      132         9                           141
   10-20% |        3       102         1                 106
 20 - 30% |                  8        87         2        97
    >=30% |                           14       274       288
          |
    Total |      135       119       102       276       632
------------------------------------------------------------

.

.
```

```
. stset followup, failure(both_levend==1)

     failure event:  both_levend == 1
obs. time interval:  (0, followup]
 exit on or before:  failure

------------------------------------------------------------------------------
     1157  total obs.
        0  exclusions
------------------------------------------------------------------------------
     1157  obs. remaining, representing
      525  failures in single record/single failure data
 11602.86  total analysis time at risk, at risk from t =         0
                             earliest observed entry t =         0
                                  last observed exit t =  16.06571

.
. stcox log_both_penkapmoll both_geslacht both_leeftijd both_bmi both_roken_baseline b
> oth_rrs both_dmduur both_hba1c both_chol_hdl_ratio both_albuminurie both_mvc both_cr
> eat, schoenfeld(sch_*) mgale(ma) scaledsch(sca_*) basesurv(base)

         failure _d:  both_levend == 1
   analysis time _t:  followup

Iteration 0:   log likelihood = -3502.2061
Iteration 1:   log likelihood = -3205.4562
Iteration 2:   log likelihood =  -3184.408
Iteration 3:   log likelihood = -3183.7722
Iteration 4:   log likelihood =   -3183.77
Refining estimates:
Iteration 0:   log likelihood =   -3183.77

Cox regression -- Breslow method for ties

No. of subjects =         1157                     Number of obs   =      1157
No. of failures =          525
Time at risk    =  11602.86106
                                                   LR chi2(12)     =    636.87
Log likelihood  =     -3183.77                     Prob > chi2     =    0.0000

-------------------------------------------------------------------------------------
                 _t | Haz. Ratio   Std. Err.      z    P>|z|     [95% Conf. Interval]
--------------------+----------------------------------------------------------------
log_both_penkapmoll |   1.090911   .1628067     0.58   0.560     .8142482    1.461578
      both_geslacht |   .8934479   .0933768    -1.08   0.281     .7279603    1.096556
      both_leeftijd |   1.107756   .0064253    17.64   0.000     1.095234    1.120421
           both_bmi |   1.002284   .0106229     0.22   0.830     .9816781    1.023322
both_roken_baseline |   1.967315   .2325963     5.72   0.000       1.5604    2.480343
           both_rrs |   .9955922   .0021007    -2.09   0.036     .9914833    .9997181
        both_dmduur |   1.015431   .0060219     2.58   0.010     1.003697    1.027303
         both_hba1c |   1.100667   .0409958     2.58   0.010     1.023179    1.184022
both_chol_hdl_ratio |   .9926371   .0315344    -0.23   0.816     .9327156    1.056408
   both_albuminurie |   1.736412   .1636795     5.85   0.000     1.443497    2.088765
           both_mvc |   1.504119   .1376422     4.46   0.000     1.257154    1.799599
         both_creat |   1.008914   .0023696     3.78   0.000      1.00428    1.013569
-------------------------------------------------------------------------------------

. estat ic

-----------------------------------------------------------------------------
       Model |    Obs    ll(null)   ll(model)     df          AIC         BIC
-------------+---------------------------------------------------------------
           . |   1157   -3502.206    -3183.77     12      6391.54    6452.183
-----------------------------------------------------------------------------
               Note:  N=Obs used in calculating BIC; see [R] BIC note

. estat concordance, gh se

         failure _d:  both_levend == 1
   analysis time _t:  followup

  Gonen and Heller's K concordance statistic

  Number of subjects (N)       =     1157

          Gonen and Heller's K =    .7753
```

```
                 Somers' D =    .5505
        Gonen's smoothed K =     .775
             Asymptotic SE =  .007343


. stcoxgof, group(10)

Goodness-of-fit test for the inclusion of design variables based on 10 quantiles of ri
> sk
(Added variables version of the Groennesby and Borgan test)

Score test                                             chi2(9)     =    13.132
                                                       Prob > chi2 =    0.1567


Likelihood-ratio test                                  LR chi2(9)  =    13.158
                                                       Prob > chi2 =    0.1556

(Table  collapsed on quantiles of linear predictor)

--------------------------------------------------------------------------------
Quantile  |
of Risk   |   Observed      Expected          z          p-Norm    Observations
----------+---------------------------------------------------------------------
        1 |          6         5.667         .14           .889             116
        2 |         14        11.832         .63           .528             116
        3 |         19         20.64       -.361           .718             116
        4 |         24         31.51      -1.338           .181             115
        5 |         36        42.081       -.937           .349             116
        6 |         49        56.325       -.976           .329             116
        7 |         75        65.662       1.152           .249             115
        8 |         88        73.999       1.628           .104             116
        9 |        102        88.435       1.442           .149             116
       10 |        112       128.849      -1.484           .138             115
          |
    Total |        525           525                                       1157
--------------------------------------------------------------------------------

. somersd _t invhr if _st==1, cenind(censind) tdist transf(c)
Somers' D with variable: _t
Transformation: Harrell's c
Valid observations: 1157
Degrees of freedom: 1156

Symmetric 95% CI for Harrell's c
------------------------------------------------------------------------------
             |              Jackknife
          _t |      Coef.   Std. Err.      t    P>|t|     [95% Conf. Interval]
-------------+----------------------------------------------------------------
       invhr |   .7968475   .0094941    83.93   0.000     .7782198    .8154751
------------------------------------------------------------------------------

. drop hr invhr censind

. str2ph stcox log_both_penkapmoll both_geslacht both_leeftijd both_bmi both_roken_bas
> eline both_rrs both_dmduur both_hba1c both_chol_hdl_ratio both_albuminurie both_mvc
> both_creat, bootreps(1000) adjust
..........

R^2 (explained variation): Cox model

   Obs    Events   Adj. R^2    Boot. SE   95% conf. interval
------------------------------------------------------------
  1157      525    0.581738    0.029508   0.529875  0.646096
------------------------------------------------------------

.
```

```
. stset followup, failure(both_levend==1)

     failure event:  both_levend == 1
obs. time interval:  (0, followup]
 exit on or before:  failure

------------------------------------------------------------------------------
     1157  total obs.
        0  exclusions
------------------------------------------------------------------------------
     1157  obs. remaining, representing
      525  failures in single record/single failure data
 11602.86  total analysis time at risk, at risk from t =         0
                             earliest observed entry t =         0
                                  last observed exit t =  16.06571

.
. stcox both_geslacht both_leeftijd both_bmi both_roken_baseline both_rrs both_dmduur
> both_hba1c both_chol_hdl_ratio both_albuminurie both_mvc both_creat, schoenfeld(sch_
> *) mgale(ma) scaledsch(sca_*) basesurv(base)

         failure _d:  both_levend == 1
   analysis time _t:  followup

Iteration 0:   log likelihood = -3502.2061
Iteration 1:   log likelihood = -3205.4302
Iteration 2:   log likelihood =  -3184.529
Iteration 3:   log likelihood = -3183.9427
Iteration 4:   log likelihood = -3183.9408
Refining estimates:
Iteration 0:   log likelihood = -3183.9408

Cox regression -- Breslow method for ties

No. of subjects =         1157                     Number of obs   =      1157
No. of failures =          525
Time at risk    =  11602.86106
                                                   LR chi2(11)     =    636.53
Log likelihood  =   -3183.9408                     Prob > chi2     =    0.0000

-------------------------------------------------------------------------------------
                 _t | Haz. Ratio   Std. Err.      z    P>|z|     [95% Conf. Interval]
--------------------+----------------------------------------------------------------
      both_geslacht |   .9103487   .0905464    -0.94   0.345     .7491078    1.106296
      both_leeftijd |   1.108599   .0062711    18.23   0.000     1.096376    1.120959
           both_bmi |   1.001225   .0104732     0.12   0.907      .980907    1.021964
both_roken_baseline |   1.975683   .2333264     5.77   0.000     1.567441    2.490253
           both_rrs |   .9956472   .0020959    -2.07   0.038     .9915478    .9997635
        both_dmduur |   1.015652   .0060095     2.62   0.009     1.003942    1.027499
         both_hba1c |    1.09693   .0403754     2.51   0.012     1.020583    1.178989
both_chol_hdl_ratio |   .9933504   .0315665    -0.21   0.834     .9333686    1.057187
   both_albuminurie |   1.733184   .1632943     5.84   0.000     1.440946    2.084691
           both_mvc |   1.505557   .1377876     4.47   0.000     1.258334    1.801352
         both_creat |   1.009528   .0021308     4.49   0.000      1.00536    1.013713
-------------------------------------------------------------------------------------

. estat ic

-----------------------------------------------------------------------------
       Model |    Obs    ll(null)   ll(model)     df          AIC         BIC
-------------+---------------------------------------------------------------
           . |   1157   -3502.206   -3183.941     11     6389.882    6445.471
-----------------------------------------------------------------------------
               Note:  N=Obs used in calculating BIC; see [R] BIC note

. estat concordance, gh se

         failure _d:  both_levend == 1
   analysis time _t:  followup

  Gonen and Heller's K concordance statistic

  Number of subjects (N)       =     1157

          Gonen and Heller's K =    .7753
                     Somers' D =    .5507
```

```
           Gonen's smoothed K =    .7751
                Asymptotic SE =  .007347


. stcoxgof, group(10)

Goodness-of-fit test for the inclusion of design variables based on 10 quantiles of ri
> sk
(Added variables version of the Groennesby and Borgan test)

Score test                                          chi2(9)     =    13.562
                                                    Prob > chi2 =    0.1388


Likelihood-ratio test                               LR chi2(9)  =    13.881
                                                    Prob > chi2 =    0.1266

(Table  collapsed on quantiles of linear predictor)

--------------------------------------------------------------------------------
Quantile  |
of Risk   |   Observed      Expected          z          p-Norm    Observations
----------+---------------------------------------------------------------------
        1 |          6         5.617        .162           .872            116
        2 |         14        11.834         .63           .529            116
        3 |         20        20.595       -.131           .896            116
        4 |         21        31.437      -1.861           .063            115
        5 |         37        42.553       -.851           .395            116
        6 |         51        55.758       -.637           .524            116
        7 |         75        63.958       1.381           .167            115
        8 |         87         75.31       1.347           .178            116
        9 |        102        89.086       1.368           .171            116
       10 |        112       128.853      -1.485           .138            115
          |
    Total |        525           525                                      1157
--------------------------------------------------------------------------------


. somersd _t invhr if _st==1, cenind(censind) tdist transf(c)
Somers' D with variable: _t
Transformation: Harrell's c
Valid observations: 1157
Degrees of freedom: 1156

Symmetric 95% CI for Harrell's c
------------------------------------------------------------------------------
             |              Jackknife
          _t |      Coef.   Std. Err.      t    P>|t|     [95% Conf. Interval]
-------------+----------------------------------------------------------------
       invhr |   .7967545   .0094953    83.91   0.000     .7781245    .8153844
------------------------------------------------------------------------------

. drop hr invhr censind

. str2ph stcox both_geslacht both_leeftijd both_bmi both_roken_baseline both_rrs both_
> dmduur both_hba1c both_chol_hdl_ratio both_albuminurie both_mvc both_creat, bootreps
> (1000) adjust
..........

R^2 (explained variation): Cox model

   Obs    Events   Adj. R^2    Boot. SE   95% conf. interval
------------------------------------------------------------
  1157      525    0.582176    0.028376   0.532037  0.645368
------------------------------------------------------------

.
. idi both_levend both_geslacht both_leeftijd both_bmi both_roken_baseline both_rrs bo
> th_dmduur both_hba1c both_chol_hdl_ratio both_albuminurie both_mvc both_creat, prvar
> s(log_both_penkapmoll)

----------------------------------------------------
      IDI |     Estimate     Std. Err.       P-value
----------+-----------------------------------------
          |     -0.00004       0.00036       0.90450
----------------------------------------------------
```

```
. nri3 both_levend both_geslacht both_leeftijd both_bmi both_roken_baseline both_rrs b
> oth_dmduur both_hba1c both_chol_hdl_ratio both_albuminurie both_mvc both_creat, prva
> rs(log_both_penkapmoll) cut(10 20 30)

------------------------------------------------------------------
      NRI |     Estimate     Std. Err.            Z       P-value
----------+-------------------------------------------------------
          |      0.01550       0.00731      2.12023       0.03399
------------------------------------------------------------------

------------------------------------------------------------
both_leve |
nd and    |
Establish |
ed risk   |    Established risk factors + new predictors
factors   |     <10%    10-20%  20 - 30%     >=30%     Total
----------+-------------------------------------------------
1         |
     <10% |       19                                      19
   10-20% |        1        17         1                  19
 20 - 30% |                           19                  19
    >=30% |                            1       467       468
          |
    Total |       20        17        21       467       525
----------+-------------------------------------------------
0         |
     <10% |      202         3                           205
   10-20% |        5       102                           107
 20 - 30% |                  6        75         2        83
    >=30% |                            3       234       237
          |
    Total |      207       111        78       236       632
------------------------------------------------------------
```

```
. stepwise, pr(.2): stcox log_both_penkapmoll both_geslacht both_leeftijd both_bmi bot
> h_roken_baseline both_rrs both_dmduur both_hba1c both_chol_hdl_ratio both_albuminuri
> e both_mvc both_creat
                      begin with full model
p = 0.8296 >= 0.2000  removing both_bmi
p = 0.8407 >= 0.2000  removing both_chol_hdl_ratio
p = 0.5807 >= 0.2000  removing log_both_penkapmoll
p = 0.3468 >= 0.2000  removing both_geslacht

Cox regression -- Breslow method for ties

No. of subjects =         1157                   Number of obs   =      1157
No. of failures =          525
Time at risk    =  11602.86106
                                                 LR chi2(8)      =    635.60
Log likelihood  =   -3184.4085                   Prob > chi2     =    0.0000

------------------------------------------------------------------------------------
                _t | Haz. Ratio   Std. Err.      z    P>|z|     [95% Conf. Interval]
-------------------+----------------------------------------------------------------
   both_albuminurie |   1.750008   .1629484     6.01   0.000     1.458083    2.100381
           both_mvc |   1.513878    .138194     4.54   0.000      1.26587    1.810475
      both_leeftijd |   1.107559   .0060495    18.70   0.000     1.095765     1.11948
         both_creat |   1.010068   .0019945     5.07   0.000     1.006167    1.013985
both_roken_baseline |   2.016182   .2326007     6.08   0.000      1.60816    2.527727
           both_rrs |   .9954118   .0020807    -2.20   0.028     .9913421    .9994981
        both_dmduur |   1.015499   .0059106     2.64   0.008      1.00398    1.027149
         both_hba1c |   1.096399   .0395509     2.55   0.011     1.021557    1.176723
------------------------------------------------------------------------------------

. estat ic

-----------------------------------------------------------------------------
       Model |    Obs    ll(null)   ll(model)     df          AIC         BIC
-------------+---------------------------------------------------------------
           . |   1157   -3502.206   -3184.409      8     6384.817    6425.246
-----------------------------------------------------------------------------
               Note:  N=Obs used in calculating BIC; see [R] BIC note

. estat concordance, gh se

         failure _d:  both_levend == 1
   analysis time _t:  followup

  Gonen and Heller's K concordance statistic

  Number of subjects (N)       =     1157

          Gonen and Heller's K =    .7752
                     Somers' D =    .5503
            Gonen's smoothed K =    .7749
                 Asymptotic SE =  .007352



. somersd _t invhr if _st==1, cenind(censind) tdist transf(c)
Somers' D with variable: _t
Transformation: Harrell's c
Valid observations: 1157
Degrees of freedom: 1156

Symmetric 95% CI for Harrell's c
------------------------------------------------------------------------------
             |              Jackknife
          _t |      Coef.   Std. Err.      t    P>|t|     [95% Conf. Interval]
-------------+----------------------------------------------------------------
       invhr |   .7965911   .0094825    84.01   0.000     .7779864    .8151959
------------------------------------------------------------------------------


.
```

```
. stset followup, failure(both_levend==1)

     failure event:  both_levend == 1
obs. time interval:  (0, followup]
 exit on or before:  failure

------------------------------------------------------------------------------
     1157  total obs.
        0  exclusions
------------------------------------------------------------------------------
     1157  obs. remaining, representing
      525  failures in single record/single failure data
 11602.86  total analysis time at risk, at risk from t =         0
                             earliest observed entry t =         0
                                  last observed exit t =  16.06571

.
. stepwise, pr(.2): stcox log_both_penkapmoll both_geslacht both_leeftijd both_bmi bot
> h_roken_baseline both_rrs both_dmduur both_hba1c both_chol_hdl_ratio both_albuminuri
> e both_mvc
                      begin with full model
p = 0.9631 >= 0.2000  removing both_chol_hdl_ratio
p = 0.6034 >= 0.2000  removing both_bmi

Cox regression -- Breslow method for ties

No. of subjects =         1157                     Number of obs   =      1157
No. of failures =          525
Time at risk    =  11602.86106
                                                   LR chi2(9)      =    623.30
Log likelihood  =   -3190.5561                     Prob > chi2     =    0.0000

-------------------------------------------------------------------------------------
                 _t | Haz. Ratio   Std. Err.      z    P>|z|     [95% Conf. Interval]
--------------------+----------------------------------------------------------------
log_both_penkapmoll |   1.345522   .1859814     2.15   0.032     1.026209    1.764193
      both_geslacht |   .7762213   .0731546    -2.69   0.007     .6453043    .9336983
      both_leeftijd |   1.108781   .0064126    17.85   0.000     1.096283    1.121421
   both_albuminurie |   1.801281   .1669745     6.35   0.000     1.502025     2.16016
both_roken_baseline |   1.833714    .213163     5.22   0.000     1.460099    2.302931
           both_rrs |   .9952481   .0020875    -2.27   0.023     .9911651     .999348
        both_dmduur |   1.015925    .005979     2.68   0.007     1.004274    1.027711
         both_hba1c |   1.101386   .0402924     2.64   0.008     1.025179    1.183257
           both_mvc |   1.558154   .1412287     4.89   0.000     1.304544    1.861067
-------------------------------------------------------------------------------------

. estat ic

-----------------------------------------------------------------------------
       Model |    Obs    ll(null)   ll(model)     df          AIC         BIC
-------------+---------------------------------------------------------------
           . |   1157   -3502.206   -3190.556      9     6399.112    6444.594
-----------------------------------------------------------------------------
               Note:  N=Obs used in calculating BIC; see [R] BIC note

. estat concordance, gh se

         failure _d:  both_levend == 1
   analysis time _t:  followup

  Gonen and Heller's K concordance statistic

  Number of subjects (N)       =     1157

          Gonen and Heller's K =    .7749
                     Somers' D =    .5498
            Gonen's smoothed K =    .7747
                 Asymptotic SE =  .007375


. somersd _t invhr if _st==1, cenind(censind) tdist transf(c)
Somers' D with variable: _t
Transformation: Harrell's c
Valid observations: 1157
Degrees of freedom: 1156
```

Symmetric 95% CI for Harrell's c

_t	Coef.	Jackknife Std. Err.	t	P>|t|	[95% Conf. Interval]	
invhr	.7945769	.0094933	83.70	0.000	.7759509	.8132028

.

```
. gen penka_x_creat=log_both_penkapmoll*both_creat  * penka_x_creat is de interactie tussen
creat en PENKA

. stepwise, pr(.2): stcox log_both_penkapmoll both_creat penka_x_creat both_geslacht b
> oth_leeftijd both_bmi both_roken_baseline both_rrs both_dmduur both_hba1c both_chol_
> hdl_ratio both_albuminurie both_mvc
                      begin with full model
p = 0.8790 >= 0.2000  removing both_chol_hdl_ratio
p = 0.7799 >= 0.2000  removing both_bmi

Cox regression -- Breslow method for ties

No. of subjects =         1157                     Number of obs   =      1157
No. of failures =          525
Time at risk    =  11602.86106
                                                   LR chi2(11)     =    641.87
Log likelihood  =   -3181.2725                     Prob > chi2     =    0.0000

------------------------------------------------------------------------------------
                 _t | Haz. Ratio   Std. Err.      z    P>|z|     [95% Conf. Interval]
--------------------+---------------------------------------------------------------
log_both_penkapmoll |   .4692891   .1800483    -1.97   0.049     .2212417    .9954374
         both_creat |   .9647613   .0187341    -1.85   0.065     .9287332    1.002187
      penka_x_creat |   1.008437    .003615     2.34   0.019     1.001376    1.015547
      both_geslacht |   .8480174   .0886002    -1.58   0.115     .6909902    1.040729
      both_leeftijd |   1.110374   .0064549    18.01   0.000     1.097795    1.123098
   both_albuminurie |   1.723033   .1618068     5.79   0.000     1.433372     2.07123
both_roken_baseline |   1.931269   .2292049     5.55   0.000     1.530458    2.437049
           both_rrs |   .9958855   .0021035    -1.95   0.051     .9917713    1.000017
        both_dmduur |   1.016012   .0059731     2.70   0.007     1.004372    1.027787
         both_hba1c |    1.09961    .040246     2.59   0.009     1.023492    1.181389
           both_mvc |   1.526823   .1402558     4.61   0.000     1.275252     1.82802
------------------------------------------------------------------------------------

. estat ic

-----------------------------------------------------------------------------
       Model |    Obs    ll(null)   ll(model)     df          AIC         BIC
-------------+---------------------------------------------------------------
           . |   1157   -3502.206   -3181.273     11     6384.545    6440.135
-----------------------------------------------------------------------------
               Note:  N=Obs used in calculating BIC; see [R] BIC note

. estat concordance, gh se

         failure _d:  both_levend == 1
   analysis time _t:  followup

  Gonen and Heller's K concordance statistic

  Number of subjects (N)       =     1157

          Gonen and Heller's K =    .7755
                     Somers' D =    .5511
            Gonen's smoothed K =    .7753
                 Asymptotic SE =   .00736


. somersd _t invhr if _st==1, cenind(censind) tdist transf(c)
Somers' D with variable: _t
Transformation: Harrell's c
Valid observations: 1157
Degrees of freedom: 1156

Symmetric 95% CI for Harrell's c
------------------------------------------------------------------------------
             |              Jackknife
          _t |      Coef.   Std. Err.      t    P>|t|     [95% Conf. Interval]
-------------+----------------------------------------------------------------
       invhr |   .7977208   .0094437    84.47   0.000      .779192    .8162496
------------------------------------------------------------------------------

. log close
      name:  All causes mortality
       log:  C:\Users\Groenier\Documents\Data\Diabetes\Statistiek\Arnold
PENKA\Log_Penka_20130405_all_causes_update_FU.log
```

closed on: 3 Jun 2014, 16:45:42
