# Supplementary material for: Serum Proenkephalin A Levels and Mortality After Long-Term Follow-Up in Patients with Type 2 Diabetes Mellitus (ZODIAC-32)
Source: PLoS One. 2015 Jul 28;10(7):e0133065. doi: 10.1371/journal.pone.0133065 (PMC4517864; doi:10.1371/journal.pone.0133065)
Supplement: S3 Statistical Analyses — (PDF) [file pone.0133065.s003.pdf]

```
------------------------------------------------------------------------------------------------
      name:  Cardiovasculaire mortaliteit
       log:  C:\Users\Groenier\Documents\Data\Diabetes\Statistiek\Arnold PENKA\Log_Penka_2013040
> 5_CV_mortality_update_FU.log
  log type:  text
 opened on:   4 Jun 2014, 15:42:19


. stset followup, failure(both_cvdeath_missing_0==1)

     failure event:  both_cvdeath_missing_0 == 1
obs. time interval:  (0, followup]
 exit on or before:  failure

------------------------------------------------------------------------------
     1157  total obs.
        0  exclusions
------------------------------------------------------------------------------
     1157  obs. remaining, representing
      224  failures in single record/single failure data
 11602.86  total analysis time at risk, at risk from t =         0
                             earliest observed entry t =         0
                                  last observed exit t =  16.06571

.
. stcox log_both_penkapmoll, mgale(ma) schoenfeld(sch_*) scaledsch(sca_*) basesurv(base)

         failure _d:  both_cvdeath_missing_0 == 1
   analysis time _t:  followup

Iteration 0:   log likelihood = -1502.3234
Iteration 1:   log likelihood =  -1466.698
Iteration 2:   log likelihood = -1466.6261
Refining estimates:
Iteration 0:   log likelihood = -1466.6261

Cox regression -- Breslow method for ties

No. of subjects =         1157                     Number of obs   =      1157
No. of failures =          224
Time at risk    =  11602.86106
                                                   LR chi2(1)      =     71.39
Log likelihood  =   -1466.6261                     Prob > chi2     =    0.0000

-------------------------------------------------------------------------------------
                 _t | Haz. Ratio   Std. Err.      z    P>|z|     [95% Conf. Interval]
--------------------+----------------------------------------------------------------
log_both_penkapmoll |   4.994133    .947266     8.48   0.000     3.443561    7.242899
-------------------------------------------------------------------------------------

. estat ic

-----------------------------------------------------------------------------
       Model |    Obs    ll(null)   ll(model)     df          AIC         BIC
-------------+---------------------------------------------------------------
           . |   1157   -1502.323   -1466.626      1     2935.252    2940.306
-----------------------------------------------------------------------------
               Note:  N=Obs used in calculating BIC; see [R] BIC note

. estat concordance, gh se

         failure _d:  both_cvdeath_missing_0 == 1
   analysis time _t:  followup

  Gonen and Heller's K concordance statistic

  Number of subjects (N)       =     1157

          Gonen and Heller's K =    .6501
                     Somers' D =    .3003
            Gonen's smoothed K =      .65
                 Asymptotic SE =   .01492



. stcoxgof, group(10)
```

```
Goodness-of-fit test for the inclusion of design variables based on 10 quantiles of risk
(Added variables version of the Groennesby and Borgan test)

Score test                                              chi2(9)     =     7.788
                                                        Prob > chi2 =    0.5556


Likelihood-ratio test                                   LR chi2(9)  =     7.443
                                                        Prob > chi2 =    0.5911

(Table  collapsed on quantiles of linear predictor)

--------------------------------------------------------------------------------
Quantile  |
of Risk   |   Observed      Expected          z           p-Norm    Observations
----------+---------------------------------------------------------------------
        1 |       13         8.367         1.602           .109            116
        2 |       14        13.676          .088            .93            117
        3 |       13        15.391         -.609           .542            117
        4 |       17        19.777         -.624           .532            129
        5 |       17        20.281         -.728           .466            113
        6 |       20         18.56          .334           .738            104
        7 |       21        26.894        -1.137           .256            120
        8 |       33        26.395         1.286           .199            110
        9 |       30         32.93         -.511            .61            116
       10 |       46         41.73          .661           .509            115
          |
    Total |      224           224                                        1157
--------------------------------------------------------------------------------


. somersd _t invhr if _st==1, cenind(censind) tdist transf(c)
Somers' D with variable: _t
Transformation: Harrell's c
Valid observations: 1157
Degrees of freedom: 1156

Symmetric 95% CI for Harrell's c
------------------------------------------------------------------------------
             |              Jackknife
          _t |      Coef.   Std. Err.      t    P>|t|     [95% Conf. Interval]
-------------+----------------------------------------------------------------
       invhr |    .653838   .0193121    33.86   0.000     .6159473    .6917287
------------------------------------------------------------------------------

. drop hr invhr censind

. str2ph stcox log_both_penkapmoll, bootreps(1000) adjust
..........

R^2 (explained variation): Cox model

   Obs    Events   Adj. R^2    Boot. SE   95% conf. interval
------------------------------------------------------------
  1157      224    0.183324    0.045377   0.102669  0.284495
------------------------------------------------------------

.
```

```
. stset followup, failure(both_cvdeath_missing_0==1)

     failure event:  both_cvdeath_missing_0 == 1
obs. time interval:  (0, followup]
 exit on or before:  failure

------------------------------------------------------------------------------
     1157  total obs.
        0  exclusions
------------------------------------------------------------------------------
     1157  obs. remaining, representing
      224  failures in single record/single failure data
 11602.86  total analysis time at risk, at risk from t =         0
                             earliest observed entry t =         0
                                  last observed exit t =  16.06571

.
. stcox log_both_penkapmoll both_geslacht both_leeftijd, schoenfeld(sch_*) mgale(ma) scaledsch(s
> ca_*) basesurv(base)

         failure _d:  both_cvdeath_missing_0 == 1
   analysis time _t:  followup

Iteration 0:   log likelihood = -1502.3234
Iteration 1:   log likelihood = -1397.6152
Iteration 2:   log likelihood = -1394.2481
Iteration 3:   log likelihood = -1394.2415
Refining estimates:
Iteration 0:   log likelihood = -1394.2415

Cox regression -- Breslow method for ties

No. of subjects =         1157                     Number of obs   =      1157
No. of failures =          224
Time at risk    =  11602.86106
                                                   LR chi2(3)      =    216.16
Log likelihood  =   -1394.2415                     Prob > chi2     =    0.0000

-------------------------------------------------------------------------------------
                 _t | Haz. Ratio   Std. Err.      z    P>|z|     [95% Conf. Interval]
--------------------+----------------------------------------------------------------
log_both_penkapmoll |   2.308361   .4893685     3.95   0.000     1.523528    3.497494
      both_geslacht |    .561977   .0782284    -4.14   0.000     .4277889    .7382569
      both_leeftijd |   1.096396   .0091356    11.04   0.000     1.078635    1.114448
-------------------------------------------------------------------------------------

. estat ic

-----------------------------------------------------------------------------
       Model |    Obs    ll(null)   ll(model)     df          AIC         BIC
-------------+---------------------------------------------------------------
           . |   1157   -1502.323   -1394.242      3     2794.483    2809.644
-----------------------------------------------------------------------------
               Note:  N=Obs used in calculating BIC; see [R] BIC note

. estat concordance, gh se

         failure _d:  both_cvdeath_missing_0 == 1
   analysis time _t:  followup

  Gonen and Heller's K concordance statistic

  Number of subjects (N)       =     1157

          Gonen and Heller's K =    .7543
                     Somers' D =    .5086
            Gonen's smoothed K =    .7541
                 Asymptotic SE =   .01168
```

```
. stcoxgof, group(10)

Goodness-of-fit test for the inclusion of design variables based on 10 quantiles of risk
(Added variables version of the Groennesby and Borgan test)

Score test                                          chi2(9)     =    15.093
                                                    Prob > chi2 =    0.0884


Likelihood-ratio test                               LR chi2(9)  =    19.572
                                                    Prob > chi2 =    0.0207

(Table  collapsed on quantiles of linear predictor)

----------------------------------------------------------------------------------
Quantile  |
of Risk   |   Observed      Expected          z           p-Norm    Observations
----------+-----------------------------------------------------------------------
        1 |         4         2.782         .73          .465           116
        2 |         8         6.136        .752          .452           116
        3 |         8          9.36       -.445          .657           116
        4 |        14        13.185        .225          .822           115
        5 |         5        19.699      -3.312          .001           116
        6 |        28         22.85       1.077          .281           116
        7 |        32        27.756        .806           .42           115
        8 |        38        33.342        .807           .42           116
        9 |        39        39.734       -.116          .907           116
       10 |        48        49.157       -.165          .869           115
          |
    Total |       224           224                                    1157
----------------------------------------------------------------------------------

. somersd _t invhr if _st==1, cenind(censind) tdist transf(c)
Somers' D with variable: _t
Transformation: Harrell's c
Valid observations: 1157
Degrees of freedom: 1156

Symmetric 95% CI for Harrell's c
--------------------------------------------------------------------------------
             |              Jackknife
          _t |      Coef.   Std. Err.      t    P>|t|     [95% Conf. Interval]
-------------+------------------------------------------------------------------
       invhr |   .7626527   .0161285    47.29   0.000     .7310083     .794297
--------------------------------------------------------------------------------

. drop hr invhr censind

. str2ph stcox log_both_penkapmoll both_geslacht both_leeftijd, bootreps(1000) adjust
.........

R^2 (explained variation): Cox model

   Obs    Events   Adj. R^2    Boot. SE   95% conf. interval
-------------------------------------------------------------
  1157      224    0.491496    0.049344   0.396141  0.591646
-------------------------------------------------------------
```

```
. stcox both_geslacht both_leeftijd, schoenfeld(sch_*) mgale(ma) scaledsch(sca_*) basesurv(base)
>

         failure _d:  both_cvdeath_missing_0 == 1
   analysis time _t:  followup

Iteration 0:   log likelihood = -1502.3234
Iteration 1:   log likelihood =  -1406.089
Iteration 2:   log likelihood = -1402.2603
Iteration 3:   log likelihood = -1402.2517
Refining estimates:
Iteration 0:   log likelihood = -1402.2517

Cox regression -- Breslow method for ties

No. of subjects =         1157                     Number of obs   =      1157
No. of failures =          224
Time at risk    =  11602.86106
                                                   LR chi2(2)      =    200.14
Log likelihood  =   -1402.2517                     Prob > chi2     =    0.0000

------------------------------------------------------------------------------
          _t | Haz. Ratio   Std. Err.      z    P>|z|     [95% Conf. Interval]
-------------+----------------------------------------------------------------
both_geslacht |   .6115514    .083807    -3.59   0.000     .4675029    .7999844
both_leeftijd |   1.108471   .0087646    13.02   0.000     1.091426    1.125784
------------------------------------------------------------------------------

. estat ic

-----------------------------------------------------------------------------
       Model |    Obs    ll(null)   ll(model)     df          AIC         BIC
-------------+---------------------------------------------------------------
           . |   1157   -1502.323   -1402.252      2     2808.503    2818.611
-----------------------------------------------------------------------------
               Note:  N=Obs used in calculating BIC; see [R] BIC note

. estat concordance, gh se

         failure _d:  both_cvdeath_missing_0 == 1
   analysis time _t:  followup

  Gonen and Heller's K concordance statistic

  Number of subjects (N)       =     1157

          Gonen and Heller's K =    .7501
                     Somers' D =    .5003
            Gonen's smoothed K =      .75
                 Asymptotic SE =   .01216


. stcoxgof, group(10)

Goodness-of-fit test for the inclusion of design variables based on 10 quantiles of risk
(Added variables version of the Groennesby and Borgan test)

Score test                                             chi2(9)     =     8.039
                                                       Prob > chi2 =    0.5302


Likelihood-ratio test                                  LR chi2(9)  =     8.416
                                                       Prob > chi2 =    0.4928

(Table  collapsed on quantiles of linear predictor)

-------------------------------------------------------------------------------
Quantile  |
of Risk   |   Observed      Expected          z          p-Norm    Observations
----------+--------------------------------------------------------------------
        1 |         3        2.718         .171          .864           116
        2 |         9        6.762         .861          .389           120
        3 |         9       11.306        -.686          .493           125
        4 |         7       12.581       -1.573          .116           102
        5 |        22       20.226         .394          .693           120
        6 |        22       26.753        -.919          .358           122
```

```
       7 |        34        28.824          .964          .335           112
       8 |        35        28.614         1.194          .233           109
       9 |        38        39.742         -.276          .782           121
      10 |        45        46.474         -.216          .829           110
         |
   Total |       224           224                                      1157
----------------------------------------------------------------------------------

. somersd _t invhr if _st==1, cenind(censind) tdist transf(c)
Somers' D with variable: _t
Transformation: Harrell's c
Valid observations: 1157
Degrees of freedom: 1156

Symmetric 95% CI for Harrell's c
--------------------------------------------------------------------------------
             |              Jackknife
          _t |      Coef.   Std. Err.      t    P>|t|     [95% Conf. Interval]
-------------+------------------------------------------------------------------
       invhr |    .756802   .0158728    47.68   0.000     .7256593    .7879448
--------------------------------------------------------------------------------

. drop hr invhr censind

. str2ph stcox both_geslacht both_leeftijd, bootreps(1000) adjust
..........

R^2 (explained variation): Cox model

   Obs    Events   Adj. R^2    Boot. SE   95% conf. interval
--------------------------------------------------------------
  1157      224    0.463645    0.048478   0.366672  0.557341
--------------------------------------------------------------

.
. idi both_cvdeath_missing_0 both_geslacht both_leeftijd, prvars(log_both_penkapmoll)

-----------------------------------------------------
      IDI |     Estimate     Std. Err.       P-value
----------+------------------------------------------
          |      0.01289       0.00371       0.00050
-----------------------------------------------------

. nri3 both_cvdeath_missing_0 both_geslacht both_leeftijd, prvars(log_both_penkapmoll) cut(10 20
>  30)

-------------------------------------------------------------------
      NRI |     Estimate     Std. Err.             Z       P-value
----------+--------------------------------------------------------
          |      0.09003       0.03670       2.45343       0.01415
-------------------------------------------------------------------

-------------------------------------------------------------
both_cvde |
ath_missi |
ng_0 and  |
Establish |
ed risk   |    Established risk factors + new predictors
factors   |     <10%    10-20%  20 - 30%     >=30%     Total
----------+--------------------------------------------------
1         |
     <10% |       13        3                             16
   10-20% |        8       26         9         1         44
 20 - 30% |                12        57        16         85
    >=30% |                           9        70         79
          |
    Total |       21       41        75        87        224
----------+--------------------------------------------------
0         |
     <10% |      269       20                            289
   10-20% |       53      233        21                  307
 20 - 30% |                42       125        29        196
    >=30% |                 3        27       111        141
          |
    Total |      322      298       173       140        933
-------------------------------------------------------------
```

```
.
. stset followup, failure(both_cvdeath_missing_0==1)

     failure event:  both_cvdeath_missing_0 == 1
obs. time interval:  (0, followup]
 exit on or before:  failure

------------------------------------------------------------------------------
     1157  total obs.
        0  exclusions
------------------------------------------------------------------------------
     1157  obs. remaining, representing
      224  failures in single record/single failure data
 11602.86  total analysis time at risk, at risk from t =         0
                             earliest observed entry t =         0
                                  last observed exit t =  16.06571

.
. stcox log_both_penkapmoll both_geslacht both_leeftijd both_bmi both_roken_baseline both_rrs bo
> th_dmduur both_hba1c both_chol_hdl_ratio both_albuminurie both_mvc both_creat, schoenfeld(sch_
> *) mgale(ma) scaledsch(sca_*) basesurv(base)

         failure _d:  both_cvdeath_missing_0 == 1
   analysis time _t:  followup

Iteration 0:   log likelihood = -1502.3234
Iteration 1:   log likelihood = -1382.3239
Iteration 2:   log likelihood = -1337.5836
Iteration 3:   log likelihood = -1332.7168
Iteration 4:   log likelihood = -1332.4724
Iteration 5:   log likelihood = -1332.4715
Iteration 6:   log likelihood = -1332.4715
Refining estimates:
Iteration 0:   log likelihood = -1332.4715

Cox regression -- Breslow method for ties

No. of subjects =         1157                     Number of obs   =      1157
No. of failures =          224
Time at risk    =  11602.86106
                                                   LR chi2(12)     =    339.70
Log likelihood  =   -1332.4715                     Prob > chi2     =    0.0000

-------------------------------------------------------------------------------------
                 _t | Haz. Ratio   Std. Err.      z    P>|z|     [95% Conf. Interval]
--------------------+----------------------------------------------------------------
log_both_penkapmoll |   1.449731    .341766     1.58   0.115     .9133163    2.301195
      both_geslacht |   .9929526   .1569129    -0.04   0.964     .7284773    1.353446
      both_leeftijd |   1.094405   .0097263    10.15   0.000     1.075507    1.113635
           both_bmi |   .9980574   .0162955    -0.12   0.905     .9666244    1.030513
both_roken_baseline |    2.45468    .431134     5.11   0.000      1.73977    3.463361
           both_rrs |   .9984279   .0032152    -0.49   0.625      .992146    1.004749
        both_dmduur |   1.019885   .0085791     2.34   0.019     1.003208    1.036839
         both_hba1c |   1.168157   .0650747     2.79   0.005     1.047329    1.302924
both_chol_hdl_ratio |   .9842554   .0470509    -0.33   0.740     .8962256    1.080932
   both_albuminurie |   2.001453   .2955607     4.70   0.000     1.498463    2.673283
           both_mvc |   1.966936    .277894     4.79   0.000      1.49118     2.59448
         both_creat |   1.013727   .0031485     4.39   0.000     1.007575    1.019917
-------------------------------------------------------------------------------------

. estat ic

-----------------------------------------------------------------------------
       Model |    Obs    ll(null)   ll(model)     df          AIC         BIC
-------------+---------------------------------------------------------------
           . |   1157   -1502.323   -1332.471     12     2688.943    2749.586
-----------------------------------------------------------------------------
               Note:  N=Obs used in calculating BIC; see [R] BIC note

. estat concordance, gh se

         failure _d:  both_cvdeath_missing_0 == 1
   analysis time _t:  followup

  Gonen and Heller's K concordance statistic

  Number of subjects (N)       =     1157
```

```
          Gonen and Heller's K =    .7836
                     Somers' D =    .5672
            Gonen's smoothed K =    .7834
                 Asymptotic SE =  .009948


. stcoxgof, group(10)

Goodness-of-fit test for the inclusion of design variables based on 10 quantiles of risk
(Added variables version of the Groennesby and Borgan test)

Score test                                            chi2(9)     =    18.908
                                                      Prob > chi2 =    0.0260


Likelihood-ratio test                                 LR chi2(9)  =    19.689
                                                      Prob > chi2 =    0.0199

(Table  collapsed on quantiles of linear predictor)

--------------------------------------------------------------------------------
Quantile  |
of Risk   |   Observed      Expected          z           p-Norm    Observations
----------+---------------------------------------------------------------------
        1 |         3         1.942         .759           .448            116
        2 |         4         4.144        -.071           .943            116
        3 |         6         7.056        -.397           .691            116
        4 |         4        11.132       -2.138           .033            115
        5 |        11        14.905       -1.012           .312            116
        6 |        21        19.688         .296           .767            116
        7 |        23        26.027        -.593           .553            115
        8 |        41        28.327        2.381           .017            116
        9 |        52        41.812        1.576           .115            116
       10 |        59        68.966         -1.2            .23            115
          |
    Total |       224           224                                       1157
--------------------------------------------------------------------------------


. somersd _t invhr if _st==1, cenind(censind) tdist transf(c)
Somers' D with variable: _t
Transformation: Harrell's c
Valid observations: 1157
Degrees of freedom: 1156

Symmetric 95% CI for Harrell's c
------------------------------------------------------------------------------
             |              Jackknife
          _t |      Coef.   Std. Err.      t    P>|t|     [95% Conf. Interval]
-------------+----------------------------------------------------------------
       invhr |   .8171077   .0138597    58.96   0.000     .7899147    .8443008
------------------------------------------------------------------------------

. drop hr invhr censind

. str2ph stcox log_both_penkapmoll both_geslacht both_leeftijd both_bmi both_roken_baseline both
> _rrs both_dmduur both_hba1c both_chol_hdl_ratio both_albuminurie both_mvc both_creat, bootreps
> (1000) adjust
..........

R^2 (explained variation): Cox model

   Obs    Events   Adj. R^2    Boot. SE   95% conf. interval
------------------------------------------------------------
  1157      224    0.668606    0.038265   0.602707  0.755487
------------------------------------------------------------
```

```
. stset followup, failure(both_cvdeath_missing_0==1)

     failure event:  both_cvdeath_missing_0 == 1
obs. time interval:  (0, followup]
 exit on or before:  failure

------------------------------------------------------------------------------
     1157  total obs.
        0  exclusions
------------------------------------------------------------------------------
     1157  obs. remaining, representing
      224  failures in single record/single failure data
 11602.86  total analysis time at risk, at risk from t =         0
                             earliest observed entry t =         0
                                  last observed exit t =  16.06571

.
. stcox both_geslacht both_leeftijd both_bmi both_roken_baseline both_rrs both_dmduur both_hba1c
>  both_chol_hdl_ratio both_albuminurie both_mvc both_creat, schoenfeld(sch_*) mgale(ma) scaleds
> ch(sca_*) basesurv(base)

         failure _d:  both_cvdeath_missing_0 == 1
   analysis time _t:  followup

Iteration 0:   log likelihood = -1502.3234
Iteration 1:   log likelihood =  -1381.744
Iteration 2:   log likelihood = -1338.4667
Iteration 3:   log likelihood = -1333.9518
Iteration 4:   log likelihood = -1333.7366
Iteration 5:   log likelihood = -1333.7359
Refining estimates:
Iteration 0:   log likelihood = -1333.7359

Cox regression -- Breslow method for ties

No. of subjects =         1157                     Number of obs   =      1157
No. of failures =          224
Time at risk    =  11602.86106
                                                   LR chi2(11)     =    337.18
Log likelihood  =   -1333.7359                     Prob > chi2     =    0.0000

--------------------------------------------------------------------------------------
                  _t | Haz. Ratio   Std. Err.      z    P>|z|     [95% Conf. Interval]
---------------------+----------------------------------------------------------------
      both_geslacht |   1.075446   .1610018     0.49   0.627     .8019694     1.44218
      both_leeftijd |   1.098093   .0095291    10.78   0.000     1.079574     1.11693
           both_bmi |    .993612   .0160774    -0.40   0.692     .9625953    1.025628
both_roken_baseline |   2.506082   .4402534     5.23   0.000     1.776075    3.536138
           both_rrs |   .9986357    .003196    -0.43   0.670     .9923912     1.00492
        both_dmduur |    1.02077    .008543     2.46   0.014     1.004163    1.037652
         both_hba1c |   1.154838   .0639008     2.60   0.009     1.036148    1.287125
both_chol_hdl_ratio |   .9869683    .047362    -0.27   0.785     .8983723    1.084302
   both_albuminurie |   1.983888    .292656     4.64   0.000     1.485768    2.649008
           both_mvc |   1.966296    .278231     4.78   0.000     1.490061    2.594741
         both_creat |   1.016467   .0026561     6.25   0.000     1.011275    1.021686
--------------------------------------------------------------------------------------

. estat ic

-----------------------------------------------------------------------------
       Model |    Obs    ll(null)   ll(model)     df          AIC         BIC
-------------+---------------------------------------------------------------
           . |   1157   -1502.323   -1333.736     11     2689.472    2745.061
-----------------------------------------------------------------------------
               Note:  N=Obs used in calculating BIC; see [R] BIC note

. estat concordance, gh se

         failure _d:  both_cvdeath_missing_0 == 1
   analysis time _t:  followup

  Gonen and Heller's K concordance statistic

  Number of subjects (N)       =     1157

          Gonen and Heller's K =    .7838
```

```
                Somers' D =    .5676
         Gonen's smoothed K =    .7836
             Asymptotic SE =      .01


. stcoxgof, group(10)

Goodness-of-fit test for the inclusion of design variables based on 10 quantiles of risk
(Added variables version of the Groennesby and Borgan test)

Score test                                            chi2(9)     =    14.169
                                                      Prob > chi2 =    0.1164


Likelihood-ratio test                                 LR chi2(9)  =    14.693
                                                      Prob > chi2 =    0.0997

(Table  collapsed on quantiles of linear predictor)

--------------------------------------------------------------------------------
Quantile  |
of Risk   |   Observed      Expected          z          p-Norm    Observations
----------+---------------------------------------------------------------------
        1 |         3         1.879         .818           .414           116
        2 |         4         4.127        -.063            .95           116
        3 |         7          7.46        -.168           .866           116
        4 |         5        10.529       -1.704           .088           115
        5 |         9        15.054        -1.56           .119           116
        6 |        20        20.684         -.15            .88           116
        7 |        28        24.108         .793           .428           115
        8 |        40        28.759        2.096           .036           116
        9 |        49        42.626         .976           .329           116
       10 |        59        68.774       -1.179           .239           115
          |
    Total |       224           224                                      1157
--------------------------------------------------------------------------------


. somersd _t invhr if _st==1, cenind(censind) tdist transf(c)
Somers' D with variable: _t
Transformation: Harrell's c
Valid observations: 1157
Degrees of freedom: 1156

Symmetric 95% CI for Harrell's c
------------------------------------------------------------------------------
             |              Jackknife
          _t |      Coef.   Std. Err.      t    P>|t|     [95% Conf. Interval]
-------------+----------------------------------------------------------------
       invhr |   .8154656   .0138665    58.81   0.000     .7882592    .8426721
------------------------------------------------------------------------------

. drop hr invhr censind

. str2ph stcox both_geslacht both_leeftijd both_bmi both_roken_baseline both_rrs both_dmduur bot
> h_hba1c both_chol_hdl_ratio both_albuminurie both_mvc both_creat, bootreps(1000) adjust
..........

R^2 (explained variation): Cox model

   Obs    Events   Adj. R^2    Boot. SE   95% conf. interval
------------------------------------------------------------
  1157      224    0.666633    0.039915   0.595443  0.752892
------------------------------------------------------------

.
. idi both_cvdeath_missing_0 both_geslacht both_leeftijd both_bmi both_roken_baseline both_rrs b
> oth_dmduur both_hba1c both_chol_hdl_ratio both_albuminurie both_mvc both_creat, prvars(log_bot
> h_penkapmoll)

----------------------------------------------------
      IDI |     Estimate     Std. Err.       P-value
----------+-----------------------------------------
          |      0.00214       0.00179       0.22970
----------------------------------------------------
```

```
. nri3 both_cvdeath_missing_0 both_geslacht both_leeftijd both_bmi both_roken_baseline both_rrs
> both_dmduur both_hba1c both_chol_hdl_ratio both_albuminurie both_mvc both_creat, prvars(log_bo
> th_penkapmoll) cut(10 20 30)

------------------------------------------------------------------
      NRI |     Estimate     Std. Err.            Z       P-value
----------+-------------------------------------------------------
          |     -0.00018       0.02244      -0.00789      0.99371
------------------------------------------------------------------

------------------------------------------------------------
both_cvde |
ath_missi |
ng_0 and  |
Establish |
ed risk   |    Established risk factors + new predictors
factors   |     <10%    10-20%  20 - 30%     >=30%     Total
----------+-------------------------------------------------
1         |
     <10% |       18                                     18
   10-20% |        3        30         4                 37
 20 - 30% |                  2        44         6       52
    >=30% |                            6       111      117
          |
    Total |       21        32        54       117      224
----------+-------------------------------------------------
0         |
     <10% |      429        21                          450
   10-20% |       20       210        14                244
 20 - 30% |                  8        82        15      105
    >=30% |                           11       123      134
          |
    Total |      449       239       107       138      933
------------------------------------------------------------
```

```
. stepwise, pr(.2): stcox log_both_penkapmoll both_geslacht both_leeftijd both_bmi both_roken_ba
> seline both_rrs both_dmduur both_hba1c both_chol_hdl_ratio both_albuminurie both_mvc both_crea
> t
                      begin with full model
p = 0.9643 >= 0.2000  removing both_geslacht
p = 0.8945 >= 0.2000  removing both_bmi
p = 0.7221 >= 0.2000  removing both_chol_hdl_ratio
p = 0.6021 >= 0.2000  removing both_rrs

Cox regression -- Breslow method for ties

No. of subjects =         1157                     Number of obs   =      1157
No. of failures =          224
Time at risk    =  11602.86106
                                                   LR chi2(8)      =    339.28
Log likelihood  =   -1332.6811                     Prob > chi2     =    0.0000

------------------------------------------------------------------------------------
                 _t | Haz. Ratio   Std. Err.      z    P>|z|     [95% Conf. Interval]
--------------------+---------------------------------------------------------------
log_both_penkapmoll |   1.436572   .3178891     1.64   0.102     .9310454    2.216583
         both_creat |   1.013841   .0028748     4.85   0.000     1.008222    1.019491
      both_leeftijd |   1.094592   .0095969    10.31   0.000     1.075943    1.113564
           both_mvc |   1.982729    .277117     4.90   0.000      1.50763    2.607545
both_roken_baseline |   2.499508   .4200159     5.45   0.000     1.798122    3.474481
   both_albuminurie |   1.969372   .2843009     4.69   0.000     1.484046    2.613414
        both_dmduur |   1.019514    .008354     2.36   0.018     1.003271     1.03602
         both_hba1c |   1.164366   .0639212     2.77   0.006     1.045588    1.296638
------------------------------------------------------------------------------------

. estat ic

-----------------------------------------------------------------------------
       Model |    Obs    ll(null)   ll(model)     df          AIC         BIC
-------------+---------------------------------------------------------------
           . |   1157   -1502.323   -1332.681      8     2681.362    2721.791
-----------------------------------------------------------------------------
               Note:  N=Obs used in calculating BIC; see [R] BIC note

. estat concordance, gh se

         failure _d:  both_cvdeath_missing_0 == 1
   analysis time _t:  followup

  Gonen and Heller's K concordance statistic

  Number of subjects (N)       =     1157

          Gonen and Heller's K =      .784
                     Somers' D =     .5681
            Gonen's smoothed K =     .7838
                 Asymptotic SE =   .009932


. somersd _t invhr if _st==1, cenind(censind) tdist transf(c)
Somers' D with variable: _t
Transformation: Harrell's c
Valid observations: 1157
Degrees of freedom: 1156

Symmetric 95% CI for Harrell's c
------------------------------------------------------------------------------
             |              Jackknife
          _t |      Coef.   Std. Err.      t    P>|t|     [95% Conf. Interval]
-------------+----------------------------------------------------------------
       invhr |   .8172988   .0137628    59.38   0.000      .790296    .8443016
------------------------------------------------------------------------------

. drop hr invhr censind

.
```

```
. stset followup, failure(both_cvdeath_missing_0==1)

     failure event:  both_cvdeath_missing_0 == 1
obs. time interval:  (0, followup]
 exit on or before:  failure

------------------------------------------------------------------------------
     1157  total obs.
        0  exclusions
------------------------------------------------------------------------------
     1157  obs. remaining, representing
      224  failures in single record/single failure data
 11602.86  total analysis time at risk, at risk from t =         0
                             earliest observed entry t =         0
                                  last observed exit t =  16.06571

.
. stepwise, pr(.2): stcox log_both_penkapmoll both_geslacht both_leeftijd both_bmi both_roken_ba
> seline both_rrs both_dmduur both_hba1c both_chol_hdl_ratio both_albuminurie both_mvc
                      begin with full model
p = 0.9641 >= 0.2000  removing both_chol_hdl_ratio
p = 0.8312 >= 0.2000  removing both_bmi
p = 0.4138 >= 0.2000  removing both_rrs

Cox regression -- Breslow method for ties

No. of subjects =         1157                     Number of obs   =      1157
No. of failures =          224
Time at risk    =  11602.86106
                                                   LR chi2(8)      =    321.83
Log likelihood  =   -1341.4095                     Prob > chi2     =    0.0000

-------------------------------------------------------------------------------------
                 _t | Haz. Ratio   Std. Err.      z    P>|z|     [95% Conf. Interval]
--------------------+----------------------------------------------------------------
log_both_penkapmoll |   2.282138   .4890231     3.85   0.000     1.499492    3.473281
      both_geslacht |   .7665787   .1096173    -1.86   0.063     .5792137    1.014553
      both_leeftijd |   1.093854   .0097683    10.05   0.000     1.074875    1.113168
   both_albuminurie |   2.124267   .3027455     5.29   0.000     1.606564    2.808797
both_roken_baseline |   2.183994    .371101     4.60   0.000     1.565368    3.047097
           both_mvc |   2.074499   .2885938     5.25   0.000     1.579423    2.724759
        both_dmduur |   1.019652   .0084308     2.35   0.019     1.003261    1.036311
         both_hba1c |   1.162389   .0639921     2.73   0.006     1.043497    1.294828
-------------------------------------------------------------------------------------

. estat ic

-----------------------------------------------------------------------------
       Model |    Obs    ll(null)   ll(model)     df          AIC         BIC
-------------+---------------------------------------------------------------
           . |   1157   -1502.323    -1341.41      8     2698.819    2739.248
-----------------------------------------------------------------------------
               Note:  N=Obs used in calculating BIC; see [R] BIC note

. estat concordance, gh se

         failure _d:  both_cvdeath_missing_0 == 1
   analysis time _t:  followup

  Gonen and Heller's K concordance statistic

  Number of subjects (N)       =     1157

          Gonen and Heller's K =    .7838
                     Somers' D =    .5676
            Gonen's smoothed K =    .7836
                 Asymptotic SE =  .009886


. somersd _t invhr if _st==1, cenind(censind) tdist transf(c)
Somers' D with variable: _t
Transformation: Harrell's c
Valid observations: 1157
Degrees of freedom: 1156
```

Symmetric 95% CI for Harrell's c

```
------------------------------------------------------------------------------
             |              Jackknife
          _t |      Coef.   Std. Err.      t    P>|t|     [95% Conf. Interval]
-------------+----------------------------------------------------------------
       invhr |   .8131161    .013861    58.66   0.000     .7859206    .8403116
------------------------------------------------------------------------------
```

```
. drop hr invhr censind
```

```
. gen penka_x_creat=log_both_penkapmoll*both_creat  * penka_x_creat is de interactive tussen
creat en PENKA

. stepwise, pr(.2): stcox log_both_penkapmoll both_creat penka_x_creat both_geslacht both_leefti
> jd both_bmi both_roken_baseline both_rrs both_dmduur both_hba1c both_chol_hdl_ratio both_album
> inurie both_mvc
                      begin with full model
p = 0.9795 >= 0.2000  removing penka_x_creat
p = 0.9643 >= 0.2000  removing both_geslacht
p = 0.8945 >= 0.2000  removing both_bmi
p = 0.7221 >= 0.2000  removing both_chol_hdl_ratio
p = 0.6021 >= 0.2000  removing both_rrs

Cox regression -- Breslow method for ties

No. of subjects =         1157                     Number of obs   =      1157
No. of failures =          224
Time at risk    =  11602.86106
                                                   LR chi2(8)      =    339.28
Log likelihood  =   -1332.6811                     Prob > chi2     =    0.0000

------------------------------------------------------------------------------------
                _t | Haz. Ratio   Std. Err.      z    P>|z|     [95% Conf. Interval]
-------------------+----------------------------------------------------------------
log_both_penkapmoll |   1.436572   .3178891     1.64   0.102     .9310454    2.216583
        both_creat |   1.013841   .0028748     4.85   0.000     1.008222    1.019491
          both_mvc |   1.982729    .277117     4.90   0.000      1.50763    2.607545
   both_albuminurie |   1.969372   .2843009     4.69   0.000     1.484046    2.613414
      both_leeftijd |   1.094592   .0095969    10.31   0.000     1.075943    1.113564
        both_hba1c |   1.164366   .0639212     2.77   0.006     1.045588    1.296638
both_roken_baseline |   2.499508   .4200159     5.45   0.000     1.798122    3.474481
       both_dmduur |   1.019514    .008354     2.36   0.018     1.003271     1.03602
------------------------------------------------------------------------------------

. estat ic

-----------------------------------------------------------------------------
       Model |    Obs    ll(null)   ll(model)     df          AIC         BIC
-------------+---------------------------------------------------------------
           . |   1157   -1502.323   -1332.681      8     2681.362    2721.791
-----------------------------------------------------------------------------
               Note:  N=Obs used in calculating BIC; see [R] BIC note

. estat concordance, gh se

         failure _d:  both_cvdeath_missing_0 == 1
   analysis time _t:  followup

  Gonen and Heller's K concordance statistic

  Number of subjects (N)       =     1157

          Gonen and Heller's K =     .784
                     Somers' D =    .5681
            Gonen's smoothed K =    .7838
                 Asymptotic SE =  .009932


. somersd _t invhr if _st==1, cenind(censind) tdist transf(c)
Somers' D with variable: _t
Transformation: Harrell's c
Valid observations: 1157
Degrees of freedom: 1156

Symmetric 95% CI for Harrell's c
------------------------------------------------------------------------------
             |              Jackknife
          _t |      Coef.   Std. Err.      t    P>|t|     [95% Conf. Interval]
-------------+----------------------------------------------------------------
       invhr |   .8172988   .0137628    59.38   0.000      .790296    .8443016
------------------------------------------------------------------------------

. drop hr invhr censind

.
. log close
```
