# Supplementary material for: Serum Proenkephalin A Levels and Mortality After Long-Term Follow-Up in Patients with Type 2 Diabetes Mellitus (ZODIAC-32)
Source: PLoS One. 2015 Jul 28;10(7):e0133065. doi: 10.1371/journal.pone.0133065 (PMC4517864; doi:10.1371/journal.pone.0133065)
Supplement: S4 Statistical Analyses — (PDF) [file pone.0133065.s004.pdf]

```
GET
  FILE='C:\Users\Groenier\Documents\Data\Diabetes\Statistiek\Arnold PENKA\
DATASET NAME DataSet1 WINDOW=FRONT.
SORT CASES  BY Both_creat_quartielen.
SPLIT FILE LAYERED BY Both_creat_quartielen.
COXREG Followup
  /STATUS=Both_Levend(1)
  /PATTERN BY log_both_penk_quartielen
  /CONTRAST (log_both_penk_quartielen)=Indicator(1)
  /METHOD=ENTER log_both_penk_quartielen
  /PLOT SURVIVAL
  /CRITERIA=PIN(.05) POUT(.10) ITERATE(20).
```

Cox Regression

```
[DataSet1] C:\Users\Groenier\Documents\Data\Diabetes\Statistiek\Arnold PEN
KA\Werkbestand_PENKA_20130405_alleen1157pr_updatemortaliteit.sav
```

Case Processing Summary

Serum creatinine baseline Quartielen			N	Percent
1	Cases available in analysis	Event[a]	109	35.9%
		Censored	195	64.1%
		Total	304	100.0%
	Cases dropped	Cases with missing values	0	0.0%
		Cases with negative time	0	0.0%
		Censored cases before the earliest event in a stratum	0	0.0%
		Total	0	0.0%
	Total		304	100.0%
2	Cases available in analysis	Event[a]	115	37.6%
		Censored	191	62.4%
		Total	306	100.0%
	Cases dropped	Cases with missing values	0	0.0%
		Cases with negative time	0	0.0%
		Censored cases before the earliest event in a stratum	0	0.0%
		Total	0	0.0%
	Total		306	100.0%
3	Cases available in analysis	Event[a]	125	47.5%
		Censored	138	52.5%
		Total	263	100.0%

Case Processing Summary

Serum creatinine baseline Ouartielen			N	Percent
	Cases dropped	Cases with missing values	0	0.0%
		Cases with negative time	0	0.0%
		Censored cases before the earliest event in a stratum	0	0.0%
		Total	0	0.0%
	Total		263	100.0%
4	Cases available in analysis	Event[a]	176	62.0%
		Censored	108	38.0%
		Total	284	100.0%
	Cases dropped	Cases with missing values	0	0.0%
		Cases with negative time	0	0.0%
		Censored cases before the earliest event in a stratum	0	0.0%
		Total	0	0.0%
	Total		284	100.0%

a. Dependent Variable: Followup

Categorical Variable Codings[a]

		Frequency	(1)	(2)	(3)
log_both_penk_quartielen[b]	1=1	287	0	0	0
	2=2	287	1	0	0
	3=3	295	0	1	0
	4=4	288	0	0	1

a. Category variable: log_both_penk_quartielen (log_both_penk_quartielen)

b. Indicator Parameter Coding

Block 0: Beginning Block

Omnibus Tests of Model Coefficients

Serum creatinine baseline Quartielen	-2 Log Likelihood
1	1178.039
2	1224.525
3	1304.203
4	1810.703

Omnibus Tests of Model Coefficients[a]

Serum creatinine baseline Quartielen	-2 Log Likelihood	Overall (score)			Change ...
		Chi-square	df	Sig.	Chi-square
1	1159.022	21.476	3	.000	19.016
2	1221.212	3.322	3	.345	3.314
3	1296.043	8.438	3	.038	8.160
4	1768.952	40.899	3	.000	41.751

Omnibus Tests of Model Coefficients[a]

Serum creatinine baseline Quartielen	Change From ...		Change From Previous Block		
	df	Sig.	Chi-square	df	Sig.
1	3	.000	19.016	3	.000
2	3	.346	3.314	3	.346
3	3	.043	8.160	3	.043
4	3	.000	41.751	3	.000

a. Beginning Block Number 1. Method = Enter

Variables in the Equation

Serum creatinine baseline Quartielen		B	SE	Wald	df
1	log_both_penk_quartielen			19.942	3
	log_both_penk_quartielen (1)	.463	.270	2.931	1
	log_both_penk_quartielen (2)	.583	.281	4.297	1
	log_both_penk_quartielen (3)	1.195	.271	19.413	1
2	log_both_penk_quartielen			3.289	3
	log_both_penk_quartielen (1)	.375	.253	2.191	1
	log_both_penk_quartielen (2)	.052	.277	.036	1
	log_both_penk_quartielen (3)	.346	.283	1.488	1
3	log_both_penk_quartielen			8.234	3
	log_both_penk_quartielen (1)	-.114	.280	.164	1
	log_both_penk_quartielen (2)	.271	.261	1.082	1
	log_both_penk_quartielen (3)	.541	.252	4.603	1
4	log_both_penk_quartielen			37.330	3
	log_both_penk_quartielen (1)	-.080	.344	.055	1
	log_both_penk_quartielen (2)	.694	.289	5.755	1
	log_both_penk_quartielen (3)	1.178	.272	18.729	1

Variables in the Equation

Serum creatinine baseline Quartielen		Sig.	Exp(B)
1	log_both_penk_quartielen	.000	
	log_both_penk_quartielen (1)	.087	1.589
	log_both_penk_quartielen (2)	.038	1.791
	log_both_penk_quartielen (3)	.000	3.304
2	log_both_penk_quartielen	.349	
	log_both_penk_quartielen (1)	.139	1.455
	log_both_penk_quartielen (2)	.850	1.054
	log_both_penk_quartielen (3)	.223	1.413
3	log_both_penk_quartielen	.041	
	log_both_penk_quartielen (1)	.685	.893
	log_both_penk_quartielen (2)	.298	1.311
	log_both_penk_quartielen (3)	.032	1.718
4	log_both_penk_quartielen	.000	
	log_both_penk_quartielen (1)	.815	.923
	log_both_penk_quartielen (2)	.016	2.001
	log_both_penk_quartielen (3)	.000	3.247

Covariate Means and Pattern Values

Serum creatinine baseline Quartielen		Mean	Pattern 1	Pattern 2	Pattern 3
1	log_both_penk_quartielen (1)	.270	.000	1.000	.000
	log_both_penk_quartielen (2)	.220	.000	.000	1.000
	log_both_penk_quartielen (3)	.161	.000	.000	.000
2	log_both_penk_quartielen (1)	.294	.000	1.000	.000
	log_both_penk_quartielen (2)	.252	.000	.000	1.000
	log_both_penk_quartielen (3)	.196	.000	.000	.000
3	log_both_penk_quartielen (1)	.255	.000	1.000	.000
	log_both_penk_quartielen (2)	.270	.000	.000	1.000
	log_both_penk_quartielen (3)	.243	.000	.000	.000
4	log_both_penk_quartielen (1)	.169	.000	1.000	.000
	log_both_penk_quartielen (2)	.282	.000	.000	1.000
	log_both_penk_quartielen (3)	.405	.000	.000	.000

Covariate Means and Pattern Values

Serum creatinine baseline Quartielen		Pattern 4
1	log_both_penk_quartielen (1)	.000
	log_both_penk_quartielen (2)	.000
	log_both_penk_quartielen (3)	1.000
2	log_both_penk_quartielen (1)	.000
	log_both_penk_quartielen (2)	.000
	log_both_penk_quartielen (3)	1.000
3	log_both_penk_quartielen (1)	.000
	log_both_penk_quartielen (2)	.000
	log_both_penk_quartielen (3)	1.000
4	log_both_penk_quartielen (1)	.000
	log_both_penk_quartielen (2)	.000
	log_both_penk_quartielen (3)	1.000

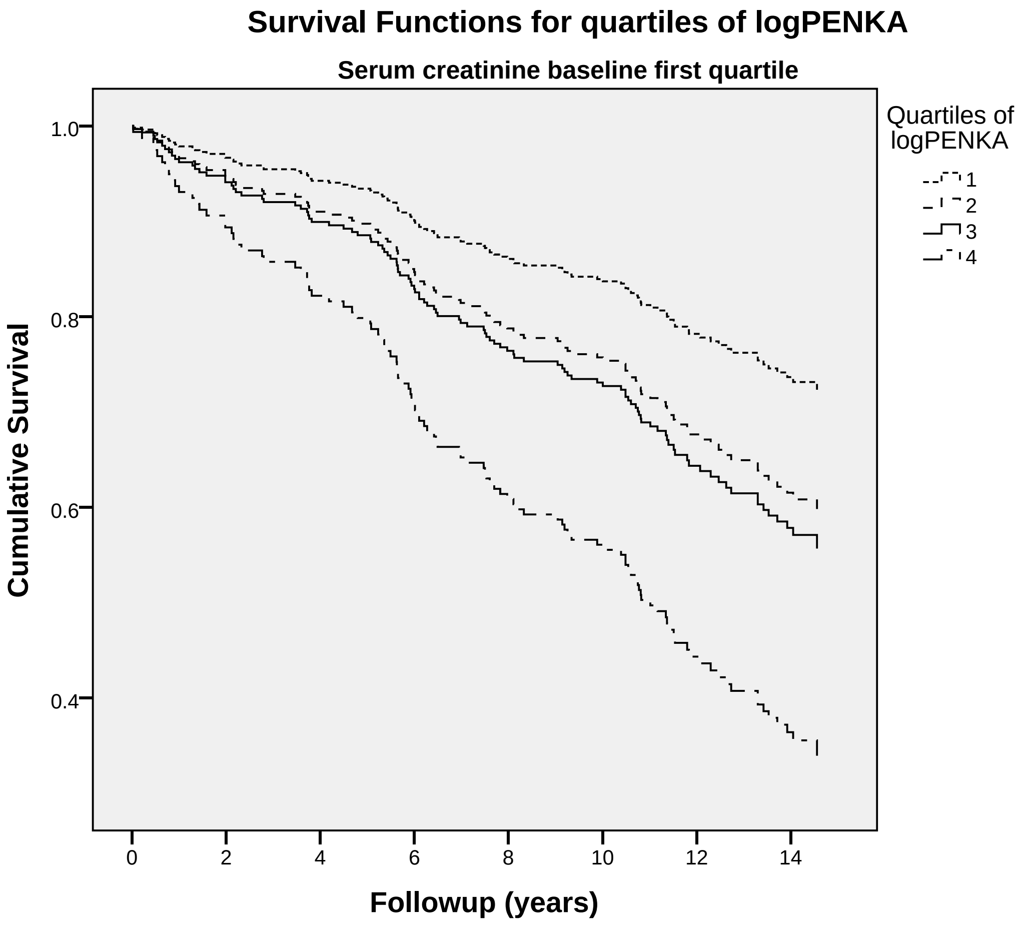

Survival Functions for quartiles of logPENKA
Serum creatinine baseline first quartile
Cumulative Survival
1.0
0.8
0.6
0.4
0
2
4
6
8
10
12
14
Followup (years)
Quartiles of logPENKA
1
2
3
4

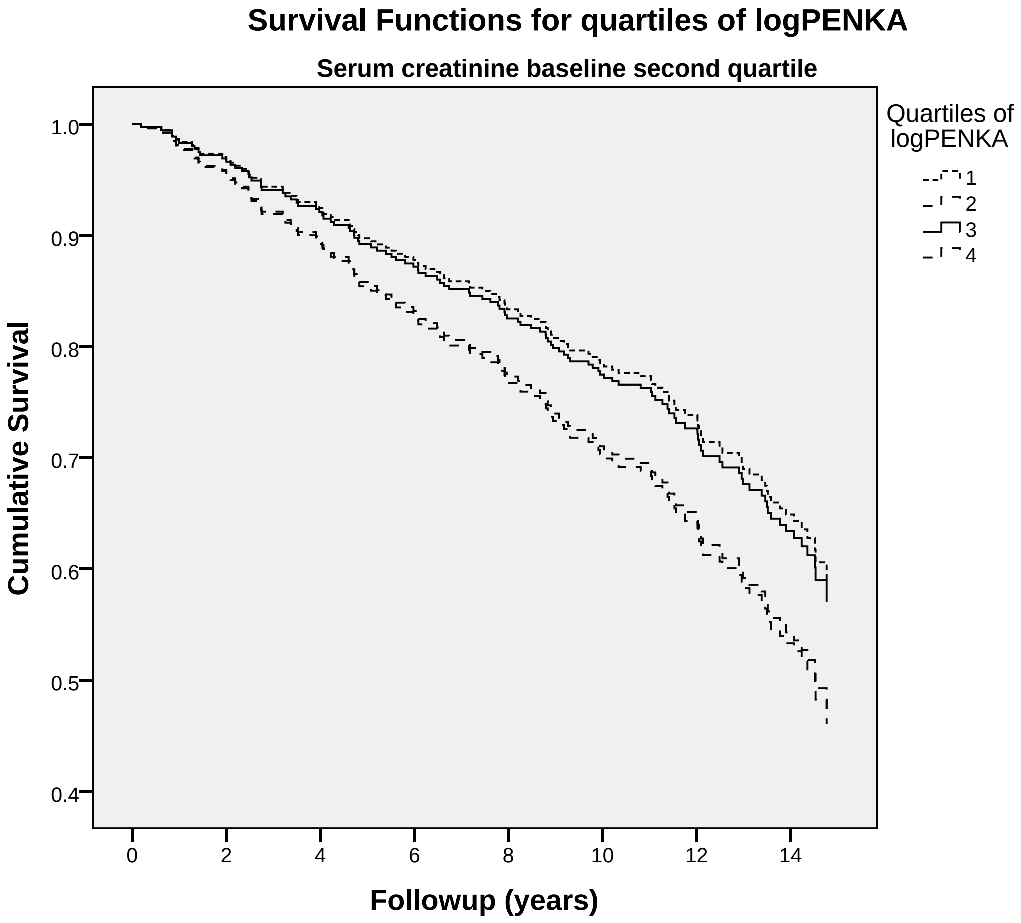

Survival Functions for quartiles of logPENKA
Serum creatinine baseline second quartile
Cumulative Survival
1.0
0.9
0.8
0.7
0.6
0.5
0.4
0
2
4
6
8
10
12
14
Followup (years)
Quartiles of logPENKA
1
2
3
4

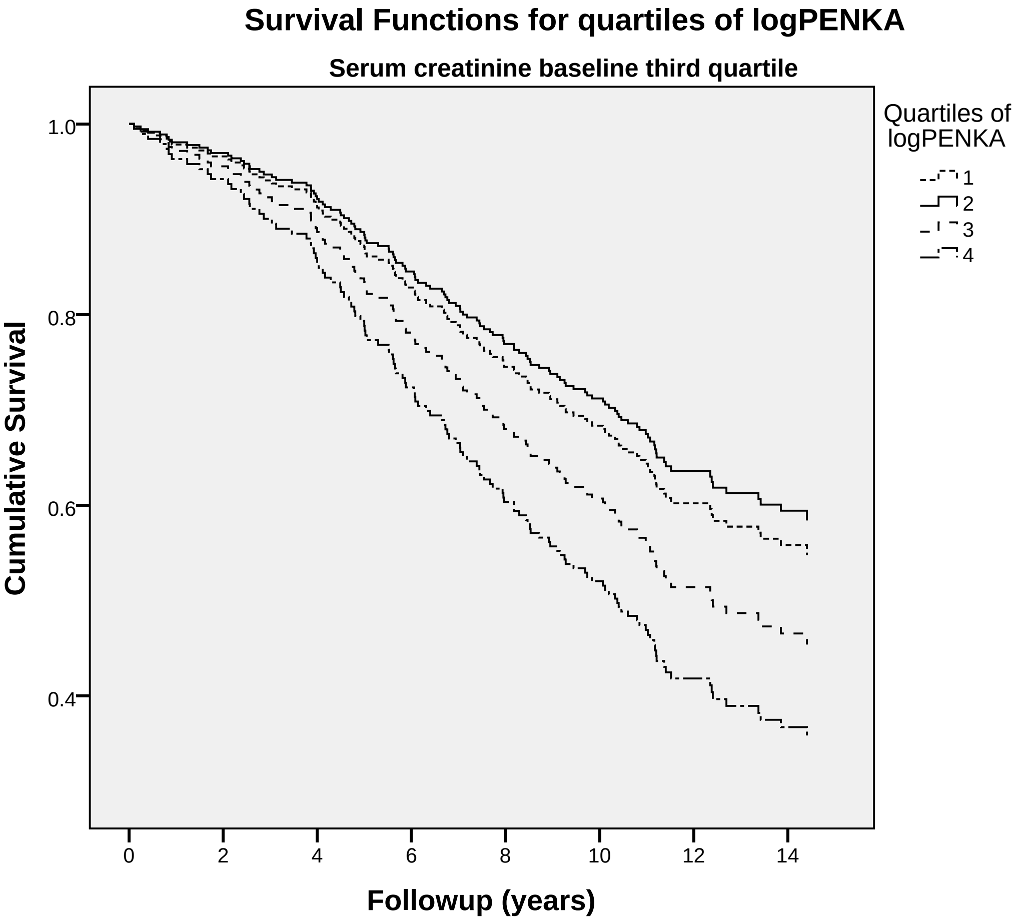
Survival Functions for quartiles of logPENKA
Serum creatinine baseline third quartile
Cumulative Survival
1.0
0.8
0.6
0.4
0
2
4
6
8
10
12
14
Followup (years)
Quartiles of logPENKA
1
2
3
4

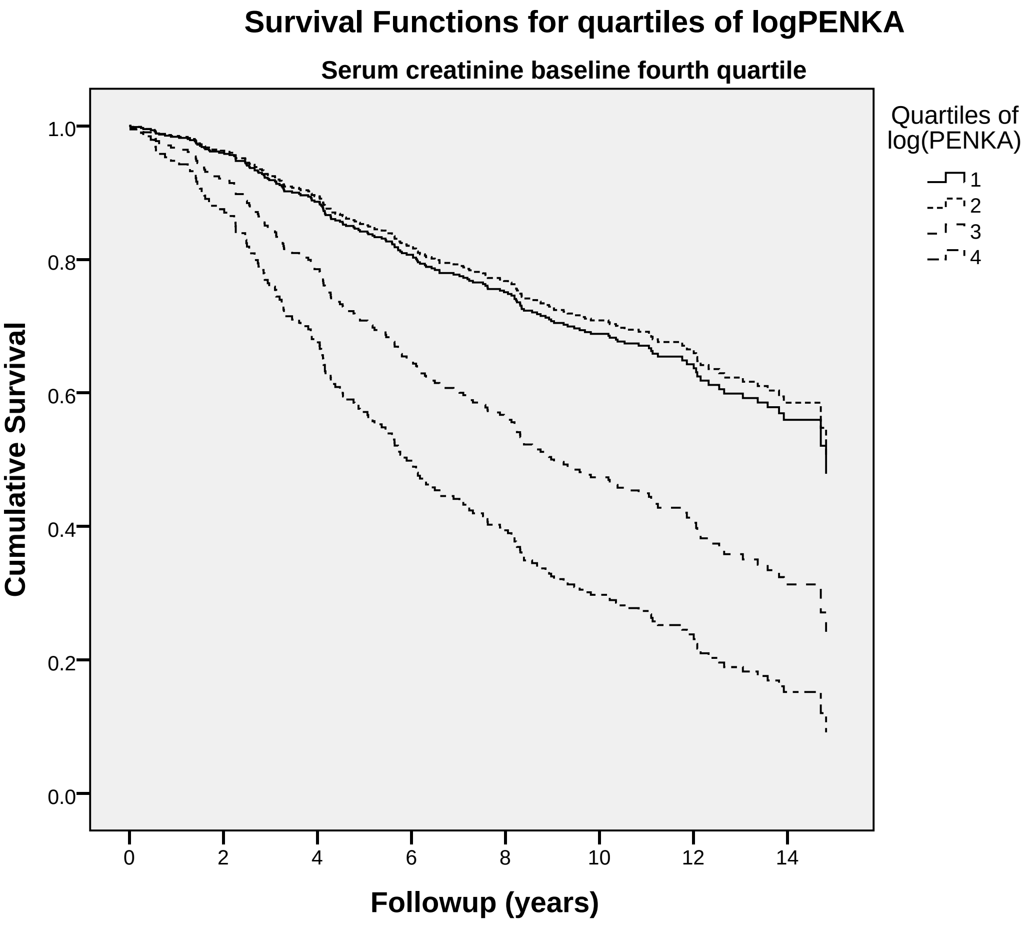


```
SORT CASES  BY log_both_penk_quartielen.
SPLIT FILE LAYERED BY log_both_penk_quartielen.
COXREG Followup
  /STATUS=Both_Levend(1)
  /PATTERN BY Both_creat_quartielen
  /CONTRAST (Both_creat_quartielen)=Indicator(1)
  /METHOD=ENTER Both_creat_quartielen
  /PLOT SURVIVAL
  /CRITERIA=PIN(.05) POUT(.10) ITERATE(20).
```

Cox Regression

[DataSet1] C:\Users\Groenier\Documents\Data\Diabetes\Statistiek\Arnold PENKA\Werkbestand_PENKA_20130405_alleen1157pr_updatemortaliteit.sav

Case Processing Summary

log_both_penk_quartielen			N	Percent
1	Cases available in analysis	Event[a]	94	32.8%
		Censored	193	67.2%
		Total	287	100.0%
	Cases dropped	Cases with missing values	0	0.0%
		Cases with negative time	0	0.0%
		Censored cases before the earliest event in a stratum	0	0.0%
		Total	0	0.0%
	Total		287	100.0%
2	Cases available in analysis	Event[a]	111	38.7%
		Censored	176	61.3%
		Total	287	100.0%
	Cases dropped	Cases with missing values	0	0.0%
		Cases with negative time	0	0.0%
		Censored cases before the earliest event in a stratum	0	0.0%
		Total	0	0.0%
	Total		287	100.0%
3	Cases available in analysis	Event[a]	134	45.4%
		Censored	161	54.6%
		Total	295	100.0%
	Cases dropped	Cases with missing values	0	0.0%
		Cases with negative time	0	0.0%
		Censored cases before the earliest event in a stratum	0	0.0%
		Total	0	0.0%
	Total		295	100.0%
4	Cases available in analysis	Event[a]	186	64.6%
		Censored	102	35.4%
		Total	288	100.0%
	Cases dropped	Cases with missing values	0	0.0%
		Cases with negative time	0	0.0%
		Censored cases before the earliest event in a stratum	0	0.0%
		Total	0	0.0%
	Total		288	100.0%

a. Dependent Variable: Followup

Categorical Variable Codings[a]

		Frequency	(1)	(2)	(3)
Both_creat_quartielen[b]	1=1	304	0	0	0
	2=2	306	1	0	0
	3=3	263	0	1	0
	4=4	284	0	0	1

a. Category variable: Both_creat_quartielen (Serum creatinine baseline Quartielen)

b. Indicator Parameter Coding

Block 0: Beginning Block

Omnibus Tests of Model Coefficients

log_both_penk_quartielen	-2 Log Likelihood
1	1008.034
2	1171.724
3	1421.016
4	1917.389

Omnibus Tests of Model Coefficients[a]

log_both_penk_quartielen	-2 Log Likelihood	Overall (score)			Change ...
		Chi-square	df	Sig.	Chi-square
1	1000.494	7.599	3	.055	7.540
2	1170.717	1.006	3	.800	1.006
3	1401.137	21.038	3	.000	19.879
4	1882.866	35.142	3	.000	34.523

Omnibus Tests of Model Coefficients[a]

log_both_penk_quartielen	Change From ...		Change From Previous Block		
	df	Sig.	Chi-square	df	Sig.
1	3	.057	7.540	3	.057
2	3	.800	1.006	3	.800
3	3	.000	19.879	3	.000
4	3	.000	34.523	3	.000

a. Beginning Block Number 1. Method = Enter

Variables in the Equation

log_both_penk_quartielen		B	SE	Wald	df	Sig.
1	Both_creat_quartielen			7.379	3	.061
	Both_creat_quartielen(1)	.345	.278	1.543	1	.214
	Both_creat_quartielen(2)	.685	.278	6.074	1	.014
	Both_creat_quartielen(3)	.658	.318	4.274	1	.039
2	Both_creat_quartielen			1.003	3	.801
	Both_creat_quartielen(1)	.244	.245	.992	1	.319
	Both_creat_quartielen(2)	.124	.273	.206	1	.650
	Both_creat_quartielen(3)	.115	.300	.146	1	.702
3	Both_creat_quartielen			19.931	3	.000
	Both_creat_quartielen(1)	-.196	.280	.490	1	.484
	Both_creat_quartielen(2)	.375	.264	2.027	1	.155
	Both_creat_quartielen(3)	.789	.247	10.228	1	.001
4	Both_creat_quartielen			32.756	3	.000
	Both_creat_quartielen(1)	-.527	.276	3.641	1	.056
	Both_creat_quartielen(2)	.011	.244	.002	1	.965
	Both_creat_quartielen(3)	.649	.214	9.234	1	.002

Variables in the Equation

log_both_penk_quartielen		Exp(B)
1	Both_creat_quartielen	
	Both_creat_quartielen(1)	1.412
	Both_creat_quartielen(2)	1.984
	Both_creat_quartielen(3)	1.931
2	Both_creat_quartielen	
	Both_creat_quartielen(1)	1.277
	Both_creat_quartielen(2)	1.132
	Both_creat_quartielen(3)	1.122
3	Both_creat_quartielen	
	Both_creat_quartielen(1)	.822
	Both_creat_quartielen(2)	1.455
	Both_creat_quartielen(3)	2.201
4	Both_creat_quartielen	
	Both_creat_quartielen(1)	.590
	Both_creat_quartielen(2)	1.011
	Both_creat_quartielen(3)	1.914

Covariate Means and Pattern Values

log_both_penk_quartielen		Mean	Pattern			
			1	2	3	4
1	Both_creat_quartielen(1)	.275	.000	1.000	.000	.000
	Both_creat_quartielen(2)	.213	.000	.000	1.000	.000
	Both_creat_quartielen(3)	.143	.000	.000	.000	1.000
2	Both_creat_quartielen(1)	.314	.000	1.000	.000	.000
	Both_creat_quartielen(2)	.233	.000	.000	1.000	.000
	Both_creat_quartielen(3)	.167	.000	.000	.000	1.000
3	Both_creat_quartielen(1)	.261	.000	1.000	.000	.000
	Both_creat_quartielen(2)	.241	.000	.000	1.000	.000
	Both_creat_quartielen(3)	.271	.000	.000	.000	1.000
4	Both_creat_quartielen(1)	.208	.000	1.000	.000	.000
	Both_creat_quartielen(2)	.222	.000	.000	1.000	.000
	Both_creat_quartielen(3)	.399	.000	.000	.000	1.000

Survival Function for patterns 1 - 4

log_both_penk_quartielen: 1

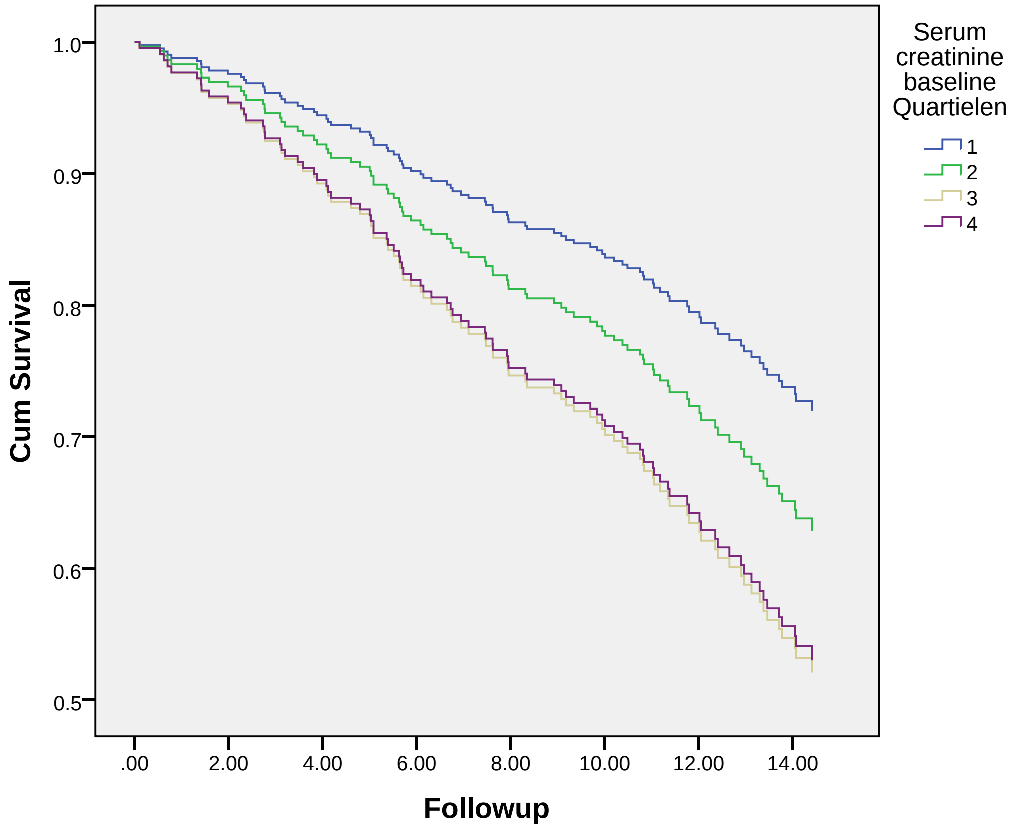

Survival Function for patterns 1 - 4

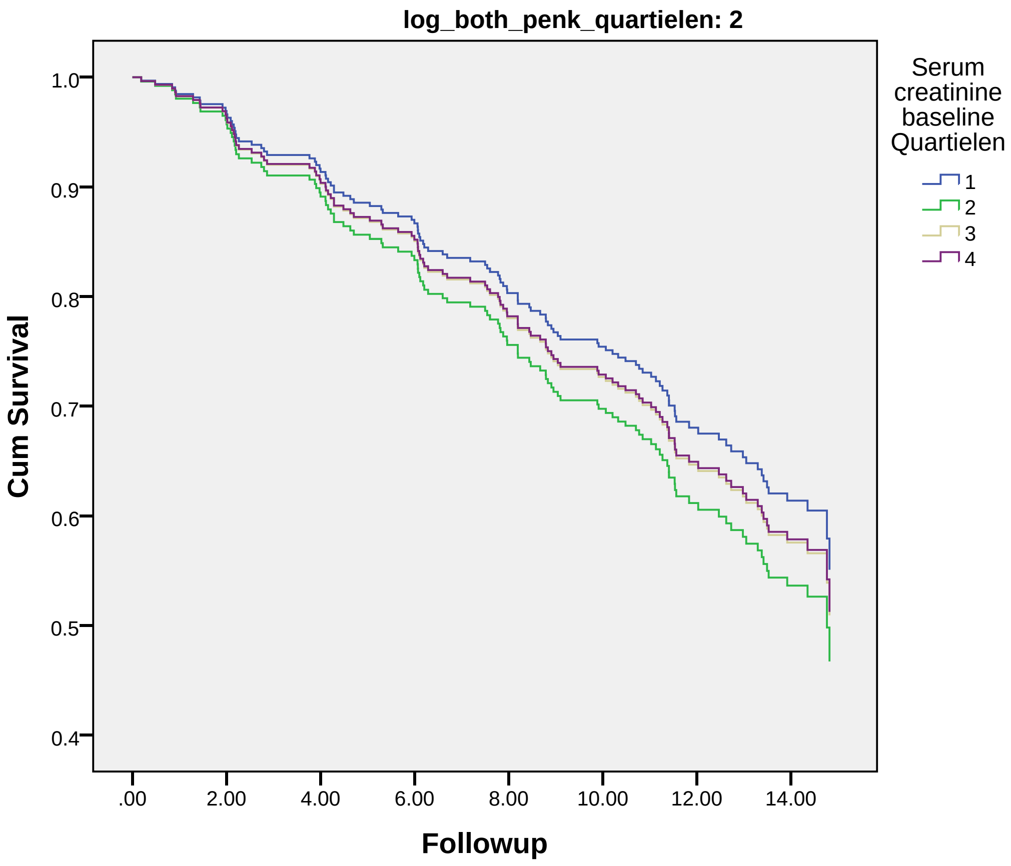

Survival Function for patterns 1 - 4

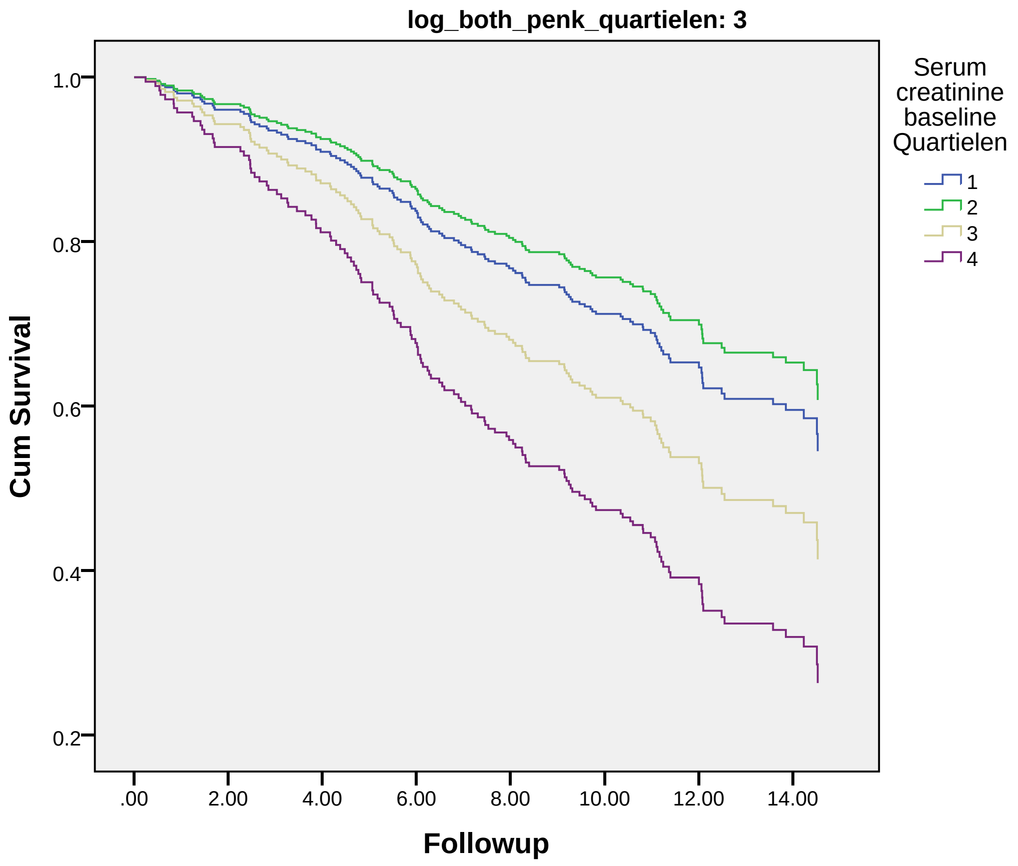

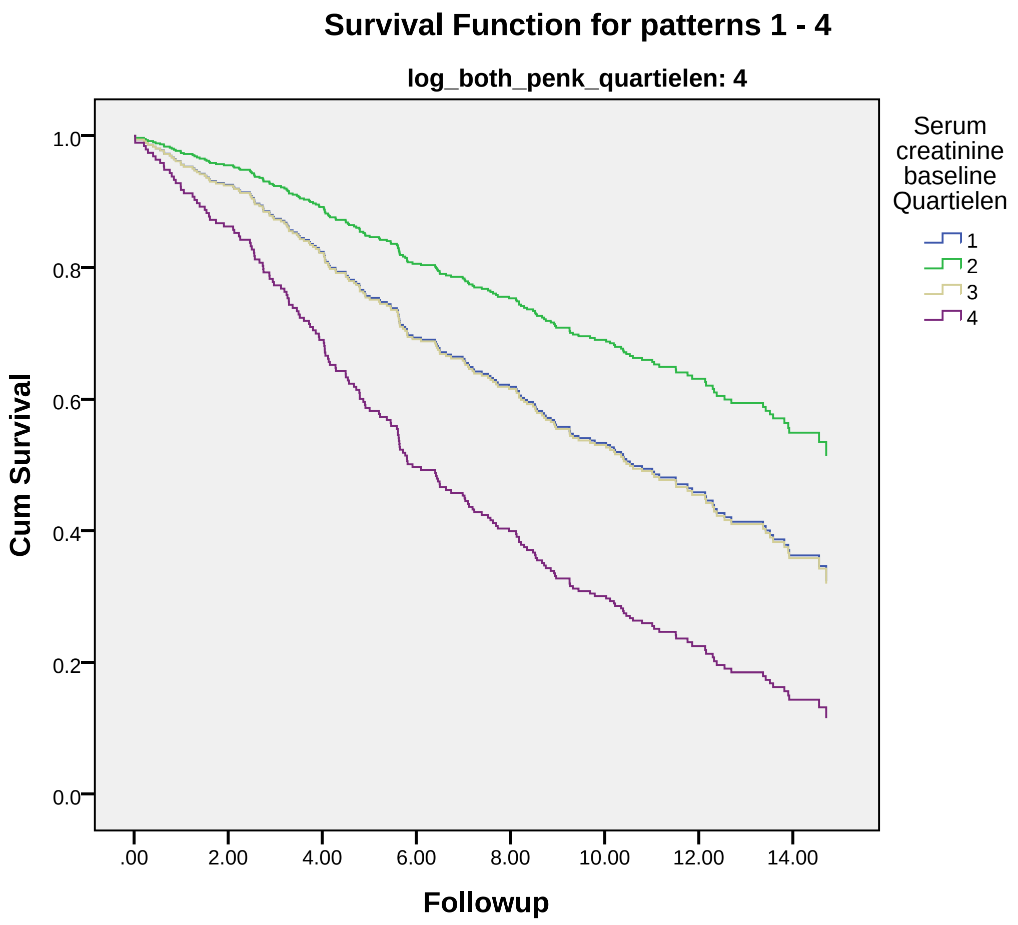

Survival Function for patterns 1 - 4
log_both_penk_quartielen: 4
Cum Survival
1.0
0.8
0.6
0.4
0.2
0.0
.00
2.00
4.00
6.00
8.00
10.00
12.00
14.00
Followup
Serum creatinine baseline Quartielen
1
2
3
4
